# Heterogeneous dynamics, robustness/fragility trade-offs, and the eradication of the macroparasitic disease, lymphatic filariasis

**DOI:** 10.1186/s12916-016-0557-y

**Published:** 2016-01-28

**Authors:** Edwin Michael, Brajendra K. Singh

**Affiliations:** Department of Biological Sciences, University of Notre Dame, Notre Dame, IN USA

**Keywords:** Vector-borne neglected tropical diseases, Lymphatic filariasis, Parasite transmission heterogeneity, Biological complexity and robustness, Parameter sloppiness, Adaptability and evolvability, Mass drug administration, Vector control, Parasite elimination programs

## Abstract

**Background:**

The current WHO-led initiative to eradicate the macroparasitic disease, lymphatic filariasis (LF), based on single-dose annual mass drug administration (MDA) represents one of the largest health programs devised to reduce the burden of tropical diseases. However, despite the advances made in instituting large-scale MDA programs in affected countries, a challenge to meeting the goal of global eradication is the heterogeneous transmission of LF across endemic regions, and the impact that such complexity may have on the effort required to interrupt transmission in all socioecological settings.

**Methods:**

Here, we apply a Bayesian computer simulation procedure to fit transmission models of LF to field data assembled from 18 sites across the major LF endemic regions of Africa, Asia and Papua New Guinea, reflecting different ecological and vector characteristics, to investigate the impacts and implications of transmission heterogeneity and complexity on filarial infection dynamics, system robustness and control.

**Results:**

We find firstly that LF elimination thresholds varied significantly between the 18 study communities owing to site variations in transmission and initial ecological parameters. We highlight how this variation in thresholds lead to the need for applying variable durations of interventions across endemic communities for achieving LF elimination; however, a major new result is the finding that filarial population responses to interventions ultimately reflect outcomes of interplays between dynamics and the biological architectures and processes that generate robustness/fragility trade-offs in parasite transmission. Intervention simulations carried out in this study further show how understanding these factors is also key to the design of options that would effectively eliminate LF from all settings. In this regard, we find how including vector control into MDA programs may not only offer a countermeasure that will reliably increase system fragility globally across all settings and hence provide a control option robust to differential locality-specific transmission dynamics, but by simultaneously reducing transmission regime variability also permit more reliable macroscopic predictions of intervention effects.

**Conclusions:**

Our results imply that a new approach, combining adaptive modelling of parasite transmission with the use of biological robustness as a design principle, is required if we are to both enhance understanding of complex parasitic infections and delineate options to facilitate their elimination effectively.

**Electronic supplementary material:**

The online version of this article (doi:10.1186/s12916-016-0557-y) contains supplementary material, which is available to authorized users.

## Background

While the current WHO-led global initiative advocating the application of annual single-dose mass drug administration (MDA) for 4–6 years to eradicate the vector-borne macroparasitic disease, lymphatic filariasis (LF), from all 73 endemic countries represents one of the largest global health programs devised to reduce the burden of tropical diseases [1, 2], a critical challenge to parasite eradication is the heterogeneous transmission of the disease across endemic regions [3–6]. We have previously shown that such environmental and geographic variability in parasite transmission between communities may reflect the impacts of significant site-specific variations in initial ecological conditions and transmission parameters [7–9]; i.e. that observed infection patterns do not merely reflect noise clouding an inherently non-spatial transmission equilibrium [10], but represent significant sensitivity to spatial and temporal variations in the key socioecological drivers of transmission across a region [8, 11]. LF transmission is further complicated by the geographic variation observed in the diversity of the primary mosquito genera implicated in parasite transmission, wherein in some agro-ecological areas *Culex* is dominant and in others, *Anopheles* or *Aedes* spp. [12–16], suggesting that site variations in vector biodiversity may also constitute a key part of the variable LF infection patterns observed across endemic regions [17].

These findings imply that spatial and temporal variability in key environmental drivers could fundamentally alter pattern-process relationships in LF transmission, and consequently lead to the likely occurrence of significant site-specific variability in parasite population response to interventions [7, 8, 11]. From a strategic perspective, these complexities imply that a single fixed time-limited global intervention strategy (as exemplified by the current WHO MDA initiative) that ignores local heterogeneities in parasite transmission and extinction dynamics is unlikely to achieve the successful elimination of this parasitic disease from all endemic regions [18, 19]. Instead, overall benefits are likely to be uneven, with re-emergence of infection and disease inevitable in those communities where transmission is not broken by the conclusion of a fixed-length intervention applied commonly everywhere [20, 21]. This observation suggests that the essentially top-down command and control management approach deployed by the WHO, which is further characterized by the selection and use of single elimination thresholds or breakpoints [7, 8, 11, 18, 22, 23], may require to be changed and made more adaptive to local transmission settings if the goal of global LF elimination is to be achieved. Alternatively, it indicates that a better understanding of how heterogeneous transmission interacts with intervention perturbations will be crucial if countermeasures robust to differential locality-specific control dynamics are to be discovered and used for achieving LF elimination reliably everywhere.

While impacts of heterogeneities in ecological and environmental factors on the transmission dynamics of vector-bone parasitic diseases, including malaria, filariasis, schistosomiasis and onchocerciasis, are a topic of growing study [5, 6, 8, 11, 22, 24], their interactions with public health interventions by contrast is only now beginning to be appreciated [11, 25–28]. Our previous work on LF transmission heterogeneity, for example, has highlighted the complex outcomes that such interactions may have for efforts aiming to achieve the elimination of parasitic disease [7–9, 11, 17]. An important finding in this regard is that while heterogeneous parasite transmission dynamics across a region may reflect strong system adaptations to site-specific environmental factors, this sensitivity to one set of localized conditions may also make a locally robustly adapted parasite system particularly fragile to perturbations that may significantly alter the variables that constrain and govern the local transmission dynamics [11]. This implies that critical trade-offs may occur between environmentally-structured transmission robustness and adaptability or even evolvability in these parasitic systems [7, 8, 11, 17, 29], suggesting that a better understanding of these “robust yet fragile” system traits, and factors that underlie these properties, will be fundamental to the development of the countermeasures needed for more effectively disrupting LF transmission from all endemic settings [7, 8, 11, 17]. Furthermore, how heterogeneous transmission dynamics interact with current drug treatment regimens to impact timelines for achieving parasite elimination in different ecological settings has also acute policy significance for the current LF elimination program, namely determining if the current WHO MDA strategy is likely to achieve the stated goal of accomplishing the elimination of this disease both regionally and globally by 2020 [7, 8, 11, 17].

In this study, our overarching goal is to examine how site-specific heterogeneity in LF transmission might affect the probability of eliminating this parasitic disease both regionally and globally using existing disease control strategies. The basis of our work is the use of a Bayesian data-model assimilation (DA) framework that facilitates both the simultaneous fitting and parameterization of vector-specific LF transmission models to parallel cross-sectional human infection and vector abundance data assembled from community field surveys [8, 9, 11, 30, 31], and the effective use of the resulting best-fitting model ensembles for undertaking numerical investigations of the effects of between-site heterogeneity on LF transmission and extinction dynamics, and the impact that this variability may have on infection outcomes in response to the mass drug and vector intervention strategies currently advocated for interrupting parasite transmission in different LF endemic settings. In addition, following recent advances in investigating the parameter structure of complex dynamical models, we also examine the parameter space and behaviour of the locally fitted models to develop new theoretical understanding regarding how such characteristics may be linked to LF transmission robustness and adaptation to the local environment, the impact that such associations may have on parasite response to perturbations, and on the ability of models to make reliable macroscopic predictions [32–34]. To be socially relevant to current control efforts, we focus on the implications that transmission heterogeneity have for two key management questions: the durations of control required for breaking LF transmission across the range of transmission intensity-vector species combinations likely to be observed in LF endemic regions; and the possible role that adding supplemental vector control measures can play in overcoming the between-site response variations that may arise from applying MDA alone.

We begin by describing our study areas and the data, followed by descriptions of the LF model and the Bayesian melding DA framework used to calibrate and fit the model to parallel community-level human infection and vector data. We then describe the modelling results focussing on how heterogeneity in transmission, parameter structure and biological robustness to extinction may interact with intervention outcomes, taking particular account of effects of variable vector species, pre-control transmission intensities, intervention coverage patterns, and the impact of supplemental vector control. We end by discussing the significance of these findings for assessing and designing the policy and management options that can best affect global LF elimination in the face of the heterogeneous dynamics and robustness trade-offs that are likely to govern local parasite transmission in typical endemic settings.

## Methods

### Data

The data used in this analysis were assembled from published pre-control cross-sectional surveys of microfilariae (mf) prevalence and mosquito abundance carried out in 18 geographically-distinct communities across the major extant LF endemic regions of Africa, Asia and Papua New Guinea. These datasets were selected on the basis that they provide human age-mf prevalence data, including break-ups of totals of individuals sampled and numbers of mf-positives out of these samples, information on the dominant prevalent vector species, and measurements of the corresponding annual mosquito biting rates (ABR) denoting the vector transmission intensity prevailing in each site. Details of the data—sample sizes and % mf-positives, along with sampling blood volumes used to assess infection prevalence, dominant vector species and ABRs—for each of the 18 survey sites are given in Table 1. Information on the drug regimen used for simulating the effects of interventions in each of these sites by MDA without/with vector control (VC) are also given, reflecting the current guidelines and use of drug combinations advocated for these sites.

**Table 1:** Description of baseline survey data. The study sites are given with the baseline sample size and microfilariae (mf) prevalence (%), blood volumes collected during the survey to test for mf positivity, annual biting rate (ABR) of vector mosquitoes, dominant vector species and drug regimen used for simulating the chemotherapeutic interventions by mass drug administration (MDA) without/with vector control (VC)

Study villages	Sample size	Blood volume (μl)	^a^Mf (%)	^b^Baseline ABR	Mosquito species (genus)	^c^Drug regimen	^d^Drug efficacies (*ω*, *ε*, *P*)	Source
Peneng	63	1,000	66.67	8,194	*An*	DEC + ALB	(55, 95, 6)	[8, 11, 78, 79]
Albulum	50	1,000	80	42,328	*An*	DEC + ALB	(55, 95, 6)	[8, 11, 78, 79]
Yauatong	131	1,000	92.37	37,052	*An*	DEC + ALB	(55, 95, 6)	[8, 11, 78, 79]
Nanaha	211	1,000	54.98	11,611	*An*	DEC + ALB	(55, 95, 6)	[8, 11, 78, 79]
Ngahmbule	346	1,000	51.16	4,346	*An*	DEC + ALB	(55, 95, 6)	[8, 11, 78, 79]
Masaika	848	100	28.61	6,184	*An*	IVM + ALB	(35, 99, 9)	[80]
Tawalani	367	100	35.72	12,850	*An*	IVM + ALB	(35, 99, 9)	[16]
Jaribuni	1,007	100	25.35	15,677	*An*	IVM + ALB	(35, 99, 9)	[81, 82]
Tingrela	699	20	63.89	4,156	*An*	IVM + ALB	(35, 99, 9)	[83]
Chiconi	245	20	58.90	10,586	*An*	IVM + ALB	(35, 99, 9)	[84]
Kingwede	825	100	3.07	1,548	*Cx*	IVM + ALB	(35, 99, 9)	[80]
Mao	546	100	27.8	25,439	*Cx*	IVM + ALB	(35, 99, 9)	[16]
Mambrui	787	100	24.99	4,964	*Cx*	IVM + ALB	(35, 99, 9)	[81, 82]
Pondicherry	1,549	20	34.74	88,500	*Cx*	DEC + ALB	(55, 95, 6)	[85]
Calcutta	861	20	26.72	115,942	*Cx*	DEC + ALB	(55, 95, 6)	[86, 87]
Vettavallam	7,976	20	22.83	100,375	*Cx*	DEC + ALB	(55, 95, 6)	[88]
Pakistan	1,443	20	31.49	1,607	*Cx*	DEC + ALB	(55, 95, 6)	[89, 90]
Jakarta	922	20	12.27	223,000	*Cx*	DEC + ALB	(55, 95, 6)	[91]

^a^All mf prevalence values were standardized to reflect sampling of 1 ml blood volumes using a transformation factor of 1.95 and 1.15, respectively, for values originally estimated using 20 or 100 μl blood volumes [49]; ^b^baseline ABR can be used to get monthly biting rate (MBR = ABR/12); ^c^the combination drug regimens are recommended by the WHO [92, 93]; ^d^the drug efficacy values are taken from [36]. *An*, *Anopheles* mosquitoes; *Cx*, *Culex* mosquitoes; drug efficacies (*ω*, *ε*, *P*) (instantaneous kill rate (%) for adult worms, instantaneous kill rate (%) for microfilariae, drug efficacy period in months); mf (%), microfilariae prevalence in percentages calculated from the number of mf-positive samples out of the total individuals sampled (sample size) in a study site. ALB, albendazole; DEC, diethylcarbamazine citrate; IVM, ivermectin

### The mathematical model of LF transmission dynamics

We employed the recently developed mosquito genus-specific transmission model of LF to carry out the modeling work in this study [7, 8, 11, 35, 36]. Briefly, the state variables of this hybrid coupled partial differential and differential equation model vary over age (*a*) and/or time (*t*), representing changes in the adult worm burden per human host (*W*(*a*, *t*)), the mf level in the human host modified to reflect infection detection in a 1 ml blood sample (*M*(*a*, *t*)), the average number of infective L3 larval stages per mosquito (*L*), and a measure of immunity (*I*(*a*, *t*)) developed by human hosts against L3 larvae. The state equations comprising this model are:

$$ \begin{array}{l}\frac{\partial W}{\partial t}+\frac{\partial W}{\partial a}=\lambda \frac{V}{H}{\psi}_1{\psi}_2h(a){L}^{*}{g}_1(I){g}_2(W)-\mu W\\ {}\frac{\partial M}{\partial t}+\frac{\partial M}{\partial a}=\alpha \phi \left(W,k\right)W-\gamma M\\ {}\frac{\partial I}{\partial t}+\frac{\partial I}{\partial a}=W-\delta I\\ {}\frac{dL}{dt}=\lambda \kappa g{\displaystyle \int \pi (a)\Big(1-f(M)}\Big)da-\sigma L-\lambda {\psi}_1L\\ {}{L}^{*}=\frac{\lambda \kappa g{\displaystyle \int \pi (a)\Big(1-f(M)}\Big)da}{\sigma +\lambda {\psi}_1}\end{array} $$

The above equations involve partial derivatives of three state variables (*W*, worm load; *M*, microfilaria intensity; *I*, immunity to acquiring new infection due to the pre-existing worm load), whereas given the faster timescale of infection dynamics in the vector compared to the human host, the infective L3-stage larval density developing in the mosquito population as a result of ingestion of mf from infected humans is modeled by an ordinary differential equation, essentially reflecting the significantly faster timescale of larval infection dynamics in the vector hosts. This allows making the simplifying assumption that the density of infective stage larvae in the vector population reaches a dynamic equilibrium (denoted by L^*^) rapidly [7, 8, 11, 37, 38]. The term *f*(*M*) describes the functional form relating the mf-L3-stage larval uptake and development in the vector population, which is famously known to differ significantly in the two major genera of mosquito vectors implicated in LF transmission [39–42], and defined as [7]:

$$ f(M)=\left[\frac{2}{{\left[1+\frac{M}{k}\left(1- \exp \left[-\frac{r}{\kappa}\right]\right)\right]}^k}-\frac{1}{{\left[1+\frac{M}{k}\left(1- \exp \left[-\frac{2r}{\kappa}\right]\right)\right]}^k}\right] $$

for mosquitoes of anopheline genus, and:

$$ f(M)={\left(1+\frac{M}{k}\left(1- \exp \left[-\frac{r}{\kappa}\right]\right)\right)}^{-k} $$

for mosquitoes of culicine genus.

In the above, *k*[=*k*_0_ + *k*_*Lin*_*M*] is the shape parameter of the negative binomial distribution indicating that mean L3 output is dependent on the distribution of mf, typically found to be overdispersed among hosts in a community [37, 43], whereas *r* and *κ* are, respectively, the rate of initial increase and the maximum level of L3 larvae that develop in each vector population. The details of the derivation of these two larval uptake and development functions are given elsewhere [7]. The terms *g*_*1*_(*I*) and *g*_*2*_(*W*) represent expressions by which acquired immunity to larval establishment, and host immunosuppression, as functions of adult worms, respectively, are included in the model [8, 11]. This basic coupled immigration-death model structure as well as recent extensions have been discussed [7, 8, 11, 37, 38]; see Additional file 1: Table S1 for the description of all the model parameters and functions.

### The Bayesian melding framework

Our strategy was essentially two-pronged: first, to integrate field observations on LF infection with simulation model outputs to undertake model calibrations and to quantify localized parasite transmission, i.e. by constraining values of transmission parameters within the bounds of data-based estimation; and second, following this to use the locally parameterized models to address the variables and questions of interest in this study, namely 1) estimation of site-specific mf age-prevalences and worm breakpoints, and 2) use of these quantities to carry out the intervention simulations described further below. We used the data-model assimilation methodology founded on the Bayesian melding (BM) algorithm to address this coupled model fitting and analyses problem [8, 11]. The BM approach is a procedure whereby all the available prior information about model inputs and outputs are “melded” together via Bayesian synthesis in order to obtain the posterior distribution of any quantity of interest that is a function of these inputs and/or outputs [31, 44]. For example, one of the priors on model output is the set of observed data; i.e. in our case the survey data on LF age-prevalence collected from each endemic community. The other output prior is the model-generated values of the state variables, such as *W* or *M*. We further specify a conditional probability distribution for observed data given the model outputs, and this yields a likelihood for each model output. Thus, the BM procedure is fundamentally a method for reconciling several sources of prior information (related to model parameters and outcomes, and data), in order to constrain the acceptable solution space of the input parameters [30, 45, 46]. In the form of the method we implemented here, we initially assigned vague or uniform prior distributions for each of the model input parameters (except for the mosquito biting rate, which was fixed to the values of the monthly biting rate (MBR; see Table 1) prevailing in each site), to reflect our initial incomplete knowledge regarding their local values, while for assessing adequacy of model outputs to data, a binomial likelihood function was constructed to capture the distribution of the observed mf age-prevalence data [8, 11, 38]. In practice, we run the dynamic model *i* times, each time drawing random input values *θ*_*i*_ for *i* = 1, … *l*, with the model producing as output the quantity of interest ϕ_*i*_, for example predictions of mf age-prevalence, for each input *θ*_*i*_. We then use the observed data, denoted by *y*, to compute a weight *w*_*i*_ for each input *θ*_*i*_: *w*_*i*_ = *L*(ϕ_*i*_). Specifically, here, *L*(ϕ_*i*_) is the likelihood of the model outputs given the observed data, *L*(ϕ_*i*_) = Prob(*y*|ϕ_*i*_). We finally use the sampling importance resampling (SIR) algorithm to resample, with replacement, from the above parameter sets with the probability of acceptance of each resample *θ*_*j =* 1,2, *… l*_ probable to its weight *w*_i_. A typical value of resamples *l* for the results presented in this paper was around 500, and these SIR parameter sets are then used to generate distributions of variables of interest from the model (e.g. age-prevalence curves, worm breakpoints), including measures of their uncertainties [8, 11]. Note that as this procedure is Monte Carlo-based, the method thus yields an ensemble of good fitting local models differing only in their parameter values as summarized by their posterior distributions.

### Numerical stability analysis for quantifying mf breakpoint and vector biting thresholds

A previously developed numerical stability analysis procedure, based on varying initial values of L^*^ to each of the SIR-selected model parameter sets or vectors, was used to calculate the distribution of mf prevalence breakpoints and threshold biting rates (TBR) expected in each study community [8, 11]. Briefly, in this procedure, we begin by progressively decreasing *V/H* from its original value to a threshold value below which the model always converges to zero mf prevalence, regardless of the values of the endemic infective larval density L^*^. The product of *λ* and this newly found *V/H* value is termed as the threshold biting rate (TBR). Once the threshold biting rate is discovered, the model at TBR will settle to either a zero (trivial attractor) or non-zero mf prevalence depending on the starting value of L^*^. Therefore, in the next step, while keeping all the model parameters unchanged, including the new *V/H*, and by starting with a very low value of L^*^ and progressively increasing it in very small step-sizes we estimate the minimum L^*^ below which the model predicts zero mf prevalence and above which the system progresses to a positive endemic infection state. The corresponding mf prevalence at this new L^*^ value is termed as the worm breakpoint in this study [7].

### Modeling intervention by mass drug administration

Intervention by MDA was modeled based on the assumption that anti-filarial treatment with a combination drug regimen acts, firstly, by killing certain fractions of the populations of adult worms and mf instantly following drug administration. These effects are incorporated into the basic model by calculating the drug-induced removal of worms and mf:

$$ \left.\begin{array}{l}W\left(a,t+dt\right)=\left(1-\omega C\left)W\right(a,t\right)\\ {}M\left(a,t+dt\right)=\left(1-\varepsilon C\right)M\left(a,t\right)\end{array}\right\}\kern1.25em \mathrm{at}\ \mathrm{time}\ t={T}_{MD{A}_i} $$

Where *dt* is a short time period since the time point ${T}_{MD{A}_i}$ when the *i*th MDA was administered. The parameters *ω* and *ε* are drug killing efficacy rates for the two life stages of the parasite, while the parameter *C* represents the MDA coverage. Apart from instantaneous killing of mf, the drug is also thought secondarily to continue to kill the newly reproduced mf by any surviving adult worms for a period of time, *P*. We model this effect as follows:

$$ \frac{\partial M\left(a,t\right)}{\partial t}+\frac{\partial M\left(a,t\right)}{\partial a}=\left(1-\varepsilon C\right)\alpha \phi \left(W\left(a,t\right),k\right)W\left(a,t\right)-\gamma M\left(a,t\right),\kern1em \mathrm{f}\mathrm{o}\mathrm{r}\ {T}_{MD{A}_i}<t\le {T}_{MD{A}_i}+P $$

#### Simulating LF MDA interventions

We simulated the effects of MDA interventions by running the model with fixed values of the three drug-related parameters (*ω*, *ε* and *P*) for MDA coverage levels ranging from 40 % to 100 %. The values of worm and mf kill rates for the two drug regimens studied here, namely diethylcarbamazine/albendazole (DEC + ALB) and ivermectin/albendazole (IVM + ALB) (Table 1), were taken from [36]. The first MDA round is implemented in the model by applying the above equations to the model vectors obtained from the baseline fits describing the pre-control worm (*W*) and mf (*M*) loads in each site, and subsequent interventions are simulated as discrete repeated pulse events acting on parasite loads resulting from each sequentially applied MDA. We investigated the impact of MDA implemented annually on the cycles or rounds of annual treatment required to reduce mf % prevalence from baseline to below the individual mf breakpoint values estimated for each SIR model vector in each site.

### Modeling vector control

We model supplemental vector control (VC) (i.e. the impact of long-lasting insecticidal nets (LLINs) or that of indoor residual spray (IRS) or the impact of the two applied in some combination) by assuming that population-level coverage of LLIN/IRS would reduce the vector biting rate to the same degree regardless of the mosquito genus present in a study site. Although efficacies of VC methods can decay over time, for example due to wear and tear of insecticidal bed nets used in the households [25, 47, 48], we do not consider this possibility here and assume for simplification that the advocated replacements of nets as well as IRS re-sprays will take place during the simulation periods examined in this paper. A full exploration of the impacts of such decay effects will be presented elsewhere. The impact of VC in this work will thus follow the modelling approach we used previously [36, 38], whereby we replace $\frac{V}{H}$ in the worm equation by the term $\left(1-{C}_V\right)\frac{V}{H}$, where *C*_*v*_ is the VC coverage in terms of the fraction of households using LLIN/IRS in a LF endemic setting.

### Model sensitivity to local conditions and feasibility of macroscopic predictions

In this exercise, we considered whether the microscopic sensitivity of LF models to local conditions may nonetheless allow general predictions of the impact of interventions at the macroscopic scale. We address this here by pooling firstly the parameter vectors from the BM fits to baseline mf age-prevalence data from each study site to create two superensembles of parameter sets: one set of parameter vectors representing the transmission dynamics across the anopheline settings in our dataset (i.e. combining the SIR vectors obtained from the five PNG and five African anopheline study sites (Table 1)); and the other for the culicine settings (containing the SIR parameter vectors from the three African and five Southeast Asian culicine sites). For each superensemble, we then ran the respective vector-specific model for the full set of ABR values (ranging from 1,500 to 230,000 bites/person/year) observed across the 18 sites, and used the resulting mf infection curves to calculate the corresponding superensemble model ABR- and TBR-associated mf % breakpoints. Only mf breakpoint values denoting a 95 % elimination probability were estimated (see below), and used as target thresholds in the intervention simulations carried out using these models.

## Results

### Model fits to baseline age-prevalence data

**Fig. 1:**
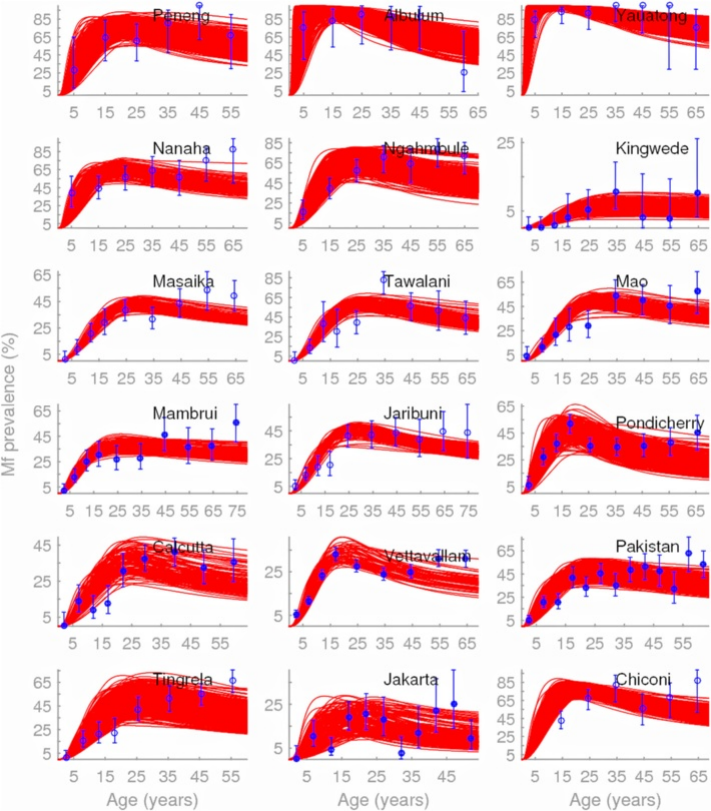
Observed and fitted microfilarial age-prevalences of lymphatic filariasis (LF) for each study site. The SIR BM model fits (red lines) to observed baseline mf prevalences in different age-groups (blue circles with binomial error-bars) from the 18 study sites investigated in this work are shown; the filled circles display the data for the culicine communities, while the open circles denote data for the anopheline communities. The age-groups are represented by the mid-point of the groups studied in each community. The study sites and details of survey data are described in Table 1. All mf prevalence values were standardized to reflect sampling of 1 ml blood volumes using a transformation factor of 1.95 and 1.15, respectively, for values originally estimated using 20 or 100 μl blood volumes [49]

The fits generated by the culicine and anopheline LF models (red curves) to the respective baseline mf prevalences in different age-groups (blue squares representing the means with lines denoting the corresponding 95 % binomial confidence intervals) from each of the 18 study sites used in this study are shown in Fig. 1. All mf prevalence values were standardized to reflect sampling of 1 ml blood volumes using a transformation factor of 1.95 and 1.15, respectively, for values originally estimated using 20 or 100 μl blood volumes [49]. Observed values, and the transformed age-profiles of mf infection showed significant differences between the study sites (Table 1; binomial generalized additive model (GAM) testing for significance of interaction between study site and mf age-prevalence patterns [50]: *χ*^2^ = 2734, *df* = 165, *p* <0.001), consistent with our previous findings that site-specific socioecologic conditions govern LF transmission patterns in the field [7, 8, 11]. The results also show that the BM-based data-model assimilation procedure is capable of reproducing the age-stratified mf prevalences consistent with observed data in each of the study communities (overall Monte Carlo *p* values >0.9 in each case (Additional file 1: Table S2)), although as expected the fits to mf age-prevalences are comparatively better for the study villages with the lowest variability in this infection measure (Fig. 1).

### Parameter values

Table 2 shows the results of a univariate Kolmogorov–Smirnov (KS) two-sample test applied to the values of prior and posterior distributions of each model parameter estimated using the Bayesian ensemble-based data-model assimilation procedure. The results show that while most of the LF model parameters exhibited variable change from initially assigned parameter values, only a few parameters pertaining to variables related to the exposure (ψ_1_, ψ_2_, H_Lin_), immunity (*c*, I_C_, S_C_) and community structure (captured indirectly by the infection aggregation parameters, e.g. k_Lin_)-related determinants of parasite transmission were consistently constrained by the site-specific data. Overall, there were also more parameters that differed from their prior values when compared across all study villages in the culicine compared to the anopheline setting (Table 2). Intriguingly, while parameters related to immunosuppression (I_C_, S_C_) were thus constrained in the villages exposed to *Anopheles* vectors, for culicine villages, by contrast, the immunity parameter most consistently constrained by site-specific data was the one associated with the strength of acquired immunity (c).

**Table 2:** Posterior changes in model parameters. Parameters whose posteriors significantly differed from their priors across all the anopheline (*An*) and culicine (*Cx*) villages are identified by the Kolmogorov–Smirnov two-sample test. The null hypothesis (H) is that priors and posteriors have the same underlying distribution. The keys are: 1, reject the null H at the 5 % significance level; and 0, do not reject the null H. Note that the parameters k_Lin_, ψ_1_, ψ_2_, H_Lin_, I_C_ and S_C_ differed from their priors across all ten or nine anopheline study villages. In the remaining culicine study villages, the parameters that differed from their priors across all eight (or seven) villages were κ, r, ψ_1_, ψ_2_, c and H_Lin_

Study villages	Spp.	λ	α	k_0_	k_Lin_	κ	r	σ	ψ_1_	ψ_2_	μ	γ	g	c	H_Lin_	V/H	I_C_	S_C_
Peneng	*An*	1	1	0	1	0	0	1	1	1	0	0	1	1	1	1	1	1
Albulum	*An*	0	1	0	1	1	0	1	1	1	1	1	0	1	1	0	1	1
Yauatong	*An*	0	1	0	1	0	1	0	0	0	1	1	0	1	1	0	1	0
Nanaha	*An*	1	0	0	1	1	1	1	1	1	0	0	0	0	0	1	1	1
Ngahmbule	*An*	0	0	0	1	0	0	1	1	1	1	0	1	1	1	0	1	1
Masaika	*An*	0	0	1	1	0	1	0	1	1	0	1	1	0	1	0	1	1
Tawalani	*An*	1	0	1	1	0	0	0	1	1	1	0	0	1	1	1	1	1
Jaribuni	*An*	0	1	0	1	0	1	1	1	1	0	0	0	0	1	0	1	1
Tingrela	*An*	0	1	0	1	0	0	1	1	1	1	0	1	1	1	0	1	1
Chiconi	*An*	0	0	0	1	1	1	1	1	1	1	1	1	1	1	0	1	1
*Sum of* An *sites*		3	5	2	10	3	5	7	9	9	6	4	5	7	9	3	10	9
Kingwede	*Cx*	0	1	1	1	0	1	1	1	1	1	1	1	1	1	0	1	0
Mao	*Cx*	1	0	0	0	0	1	0	1	1	1	0	1	1	1	1	1	0
Mambrui	*Cx*	0	1	0	0	0	1	1	1	1	0	0	0	1	1	0	1	0
Pondicherry	*Cx*	1	1	1	1	1	1	1	1	1	1	1	1	1	1	1	1	0
Calcutta	*Cx*	1	1	0	0	1	1	1	1	1	0	0	1	1	1	1	0	1
Vettavallam	*Cx*	0	1	0	1	0	1	1	1	1	1	0	1	1	0	0	1	0
Pakistan	*Cx*	1	0	0	0	1	1	1	1	1	1	0	1	1	1	1	1	1
Jakarta	*Cx*	1	1	0	1	0	1	1	1	1	1	0	0	1	1	1	0	0
*Sum of* Cx *sites*		5	6	2	4	3	8	7	8	8	6	2	6	8	7	5	6	2

**Fig. 2:**
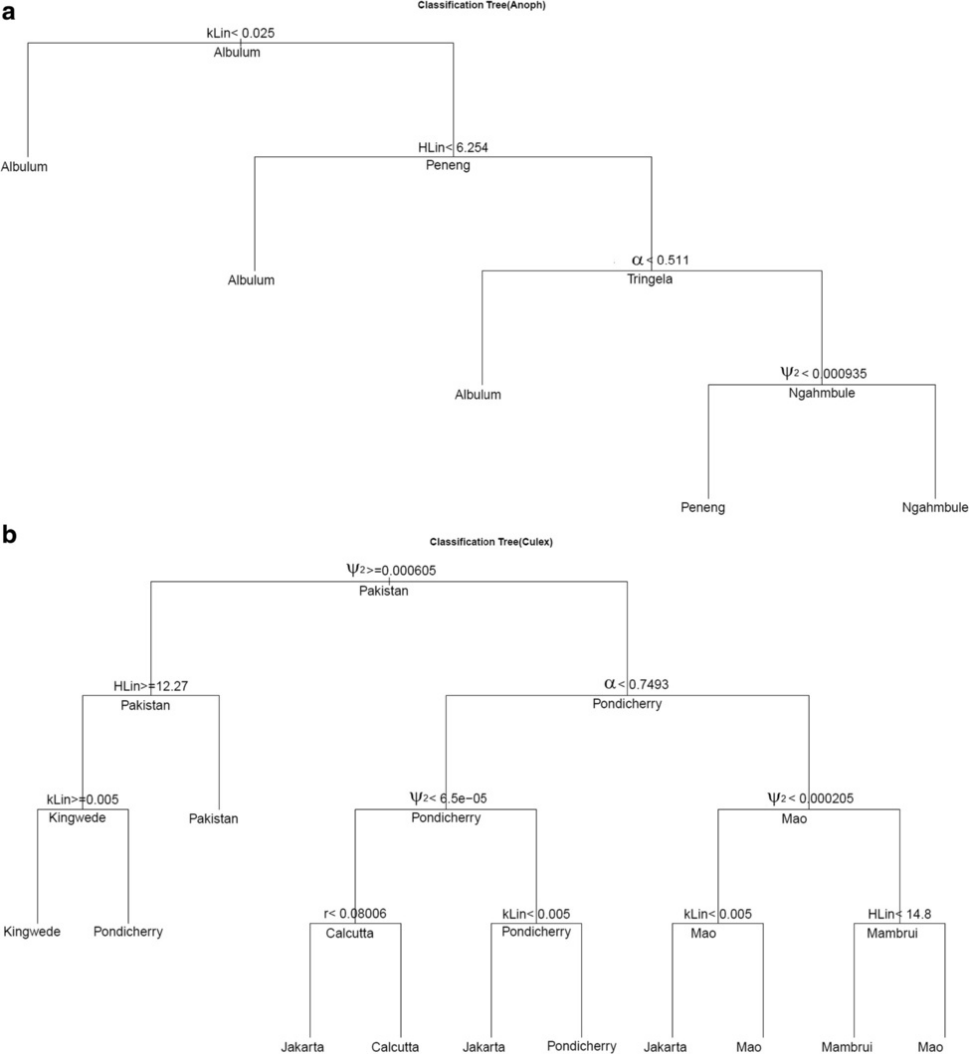
Classification tree analysis to identify model parameters that differed significantly between the present study sites. (**a**) *Anopheles* mosquitoes and (**b**) *Culex* mosquitoes. The fitted trees, stratified by mosquito species, indicate that local between-site variation in the LF infection age-patterns observed between the present study sites depended only on a few “stiff” combinations of parameters. These parameters are the H_Lin_, a threshold value used to adjust the rate at which individuals of age *a* are bitten, worm establishment rate (ψ_2_), degree of community infection aggregation (k) and worm fecundity rate (α) in both culicine (*Cx*) and anopheline (*An*) systems, and additionally the term, *r*, related to mf uptake by mosquitoes in the anopheline system. The classification trees were fitted using the rpart package in R

We used classification tree analysis next to determine which parameters differed significantly between the study communities, and therefore might underlie the between-study heterogeneity observed in the mf age-prevalence data. The fitted trees stratified by vector species are depicted in Fig. 2, and indicate that the between-site variation in LF infection age-patterns observed across the present study communities depended only on a few “stiff” combinations of parameters, again primarily those reflecting the differential exposure, degree of community infection aggregation and worm fecundity variables in both vector systems. This finding highlights that the majority of the LF model parameters may be deemed to be “sloppy” or insensitive to locally varying environmental conditions, and support recent work in systems biology suggesting that such neutral regions in multiparameter space may be a ubiquitous feature of complex systems biology models [33, 51–53].

### Threshold values and probability of LF extinction

We used the SIR-selected ensemble of parameter sets to calculate the distributions of infection breakpoints (in terms of mf %) and the vector to human transmission thresholds (the TBR) expected in each of our study sites. Mf breakpoints were furthermore estimated at both the prevailing annual biting rate (ABR) in a community as well as at the TBR value. An illustrative example, showing results from the numerical stability analysis carried out using the set of SIR parameter vectors obtained from model fits to the Peneng dataset for estimating mf % breakpoints at their TBR values is shown in Additional file 1: Figure S1. The likely existence for a distribution of system breakpoint thresholds rather than a single breakpoint in a site implied by the results shown in Additional file 1: Figure S1 also means that the probability of LF elimination or extinction will vary across the range of values of each threshold [54, 55]. Here, we use the cumulative density function (CDF) of the estimated threshold values, in conjunction with exceedance calculations [56], to quantify three mf % breakpoint threshold values denoting elimination probabilities of 50 %, 75 % and 95 % in each site in order to investigate the management trade-offs involved in their choice as intervention targets in LF elimination programs (see Additional file 1: Figure S2 for plots of the CDFs and mf % cutoffs representing these elimination probabilities in each study site).

**Fig. 3:**
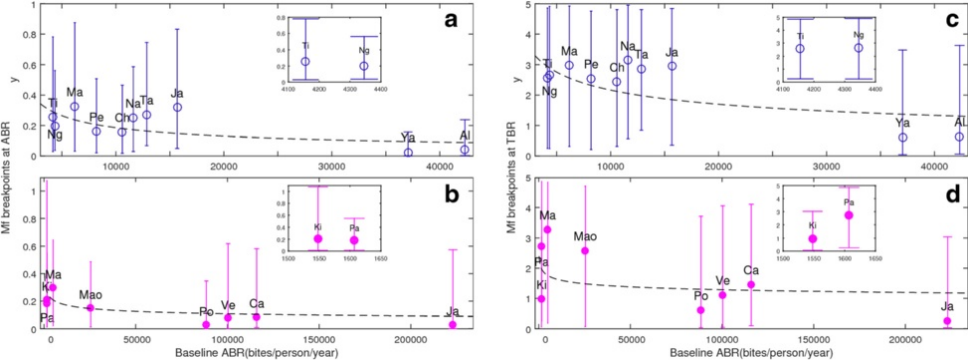
Mf breakpoints as a function of baseline community annual biting rate (ABR) and microfilaria (mf) prevalence. The mf breakpoints estimated in each site are shown as average values with 95 % CIs, calculated as the 2.5th and 97.5th percentiles of the breakpoint distribution in each site, and are plotted against the observed ABRs in each site; filled and open circles, respectively, represent values for the culicine and anopheline settings. The data in (**a**, **b**) and (**c**, **d**), respectively, represent the mf breakpoints estimated at the observed site-specific ABRs and the corresponding estimated threshold biting rates (TBRs). Both types of mf breakpoints were negatively correlated with ABR, with the fitted dashed lines indicating that overall these data follow a power-law function: *f*(*x*) = *ax* ^*b*^, with *x* representing the biting rate values on the x-axis, and *f*(*x*) the mf breakpoints on the y-axis. The term *a* is a constant while *b* is the power-law exponent, with fitted values of (*a*, *b*) as follows: (**a**) (20.54, −0.5112); (**b**) (1.335, −0.2184); (**c**) (54.25, −0.3498); and (**d**) (4.251, −0.104). All four associated *p* values were <0.01. The set of mf breakpoints plotted in each graph were calculated using the best-fitting parameter vectors obtained from model fits to the baseline mf age-profile of each study site. In the plots, individual sites are indicated by their first two letters, except for “Mao” in the culicine settings, in order to distinguish it from “Ma” used for “Mambrui”. Inset plots are provided to clarify the variations in the breakpoint values estimated for sites with approximately the same baseline ABR values, which were obscured in the respective main plots

Table 3 provides the actual numerical mf % breakpoint values signifying these probabilities at both the ABR and TBR vector transmission thresholds, and demonstrates that wide variation in their values may occur between the present study sites. Additional file 1: Table S3 presents the results of the respective binomial generalized linear model, or one-way ANOVA and Wilcoxon signed-rank tests applied to these data, and statistically support the impression from Table 1 that there existed both a significant vector species-related difference observed in the estimated values of these thresholds, with generally higher values found in the anopheline settings, as well as a statistical site-specific variation in the values of these thresholds within both the anopheline and culicine LF transmission endemic settings. The results further show that mf breakpoint values in a site are also highly dependent on the associated probability of extinction they represent, with values decreasing markedly with increasing probabilities of extinction. Figure 3, however, indicates that while the mf breakpoint values estimated at either TBR or baseline ABR are variable between the study sites, these values nonetheless may exhibit functional relationships with the baseline study ABR, with the estimated mf thresholds declining on average in a power-law fashion with increasing site-specific intensities of the host infection system input (ABR) variables in both the anopheline and culicine cases.

**Table 3:** Model-estimated worm breakpoint values for achieving the successful interruption of LF transmission in each of the study sites investigated. Breakpoints are listed in terms of % mf prevalence at three probabilities of elimination for two situations: 1) at the prevailing vector biting rates (i.e. at the observed ABRs); and 2) at the threshold biting rate (TBR) at or below which LF transmission process cannot sustain itself regardless of the level of the infection in human hosts (see text). The first set of the threshold values (at study-specific ABR) is used in modeling the impact of mass drug administration (MDA) alone, while the second set (mf breakpoint values estimated at TBR) is applied for modeling the impact when MDA is supplemented by vector control (VC)

	Mf breakpoints calculated at ABR			Mf breakpoints calculated at TBR		
Study villages	50 % EP as % mf	75 % EP as % mf	95 % EP as % mf	50 % EP as % mf	75 % EP as % mf	95 % EP as % mf
Peneng	0.203816	0.111209	0.035429	2.54805	1.603205	0.435501
Albulum	0.041834	0.018397	0.004885	0.638664	0.268399	0.094346
Yauatong	0.025019	0.01043	0.002985	0.612255	0.271548	0.066789
Nanaha	0.383165	0.207235	0.066568	3.144555	2.278285	0.919664
Ngahmbule	0.314275	0.163155	0.058476	2.65292	1.727635	0.45293
Masaika	0.547213	0.25285	0.053393	2.973785	1.555073	0.451975
Tawalani	0.377915	0.215966	0.085207	2.86278	2.006245	1.07946
Jaribuni	0.454702	0.196513	0.077864	2.946105	2.15063	1.112716
Tingrela	0.334315	0.171191	0.042786	2.56515	1.532875	0.559656
Chiconi	0.223123	0.098382	0.033768	2.437125	1.568188	0.677308
Kingwede	0.209935	0.08889	0.022236	0.972591	0.455327	0.089295
Mao	0.189285	0.117818	0.019268	2.575155	1.828288	0.384838
Mambrui	0.49878	0.25427	0.075622	3.26316	2.33081	0.885393
Pondicherry	0.028824	0.005726	0.000476	0.536544	0.146736	0.041653
Calcutta	0.092718	0.043027	0.017295	1.46021	0.762273	0.178704
Vettavallam	0.077791	0.033227	0.002706	1.110415	0.613489	0.110904
Pakistan	0.267202	0.121281	0.034034	2.72511	1.811333	0.659793
Jakarta	0.032622	0.003889	0.000171	0.191162	0.098852	0.028576

EP, elimination probability

### Impact of local transmission dynamics and breakpoints on elimination of LF

We used the locally calibrated LF models together with their corresponding site-specific mf % breakpoints to simulate the impact that locally variable LF transmission dynamics may have on the expected timelines (in the form of number of rounds of annual MDAs required) for achieving parasite extinction in each site due to the application of the two major control strategies currently proposed for eliminating LF, namely MDA alone and MDA supplemented with vector control. The analysis was carried out by subjecting each of the 500 SIR-resampled parameter sets estimated from a site to the drug regimen (i.e. either DEC + ALB or IVM + ALB) recommended for use in that setting, and assessing the number of annual cycles of MDA which would be required for all the ensemble model vectors to cross below their respective mf % breakpoint thresholds signifying 50 %, 75 % and 95 % probabilities of LF elimination (EP). Mf % breakpoint thresholds at ABR were used as targets when modelling the impact of MDA alone (Table 3), whereas breakpoint prevalence values at TBR were used when modelling the impact of including VC, as reducing the vector population will push the system towards the TBR breakpoint and hence raise mf breakpoints to their maximal values (see Additional file 1: Figure S1 and S3).

**Fig. 4:**
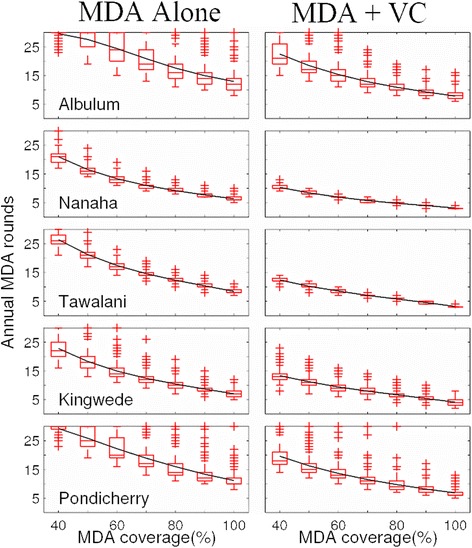
Variability in the impact of annual mass drug administration (MDA) and combined MDA plus vector control (VC) on intervention rounds in years required to eliminate LF in different endemic communities (results shown for selected study sites). The required annual MDA rounds without and with VC as a function of drug coverage (from 40 % to 100 %) are shown as box plots, with the solid horizontal line depicting the means. Supplemental use of vector control (VC) was modelled at 80 % coverage. The results are shown for mf breakpoint threshold values representing a 95 % elimination probability (see Table 3). The results for the remaining study sites are shown in Additional file 1: Figure S4 and S5. These results are from the model simulations carried out for both LF intervention scenarios using the site-specific parameter vectors that best-fitted baseline age-prevalence infection in each site (compare with Fig. 1)

Figure 4 shows the annual MDA cycles (the boxes indicating the mean and variance in the rounds) required to cross below the site-specific 95 % EP mf % thresholds quantified for a selection of our anopheline and culicine study sites (with results for the rest of the sites given in Additional file 1: Figure S4 and S5). Results are illustrated for a range of drug coverages (from 40 % to 100 %) and with and without inclusion of VC. These indicate firstly that while in general the number of years of annual MDA rounds required to achieve parasite elimination will decline with increasing drug coverage, the actual MDAs required at any given drug coverage will vary significantly between sites (Fig. 4, Additional file 1: Figure S4 and S5, Additional file 1: Table S4). Inclusion of VC, however, will not only strikingly reduce the numbers of annual MDAs needed (in some cases from decades of treatment to more feasible MDA durations (less than 10 years in general even for a drug coverage as high as 80 %)), but it will also, interestingly, reduce the variance in treatment rounds required compared to when using MDA alone (Fig. 4, Additional file 1: Figure S4 and S5).

**Fig. 5:**
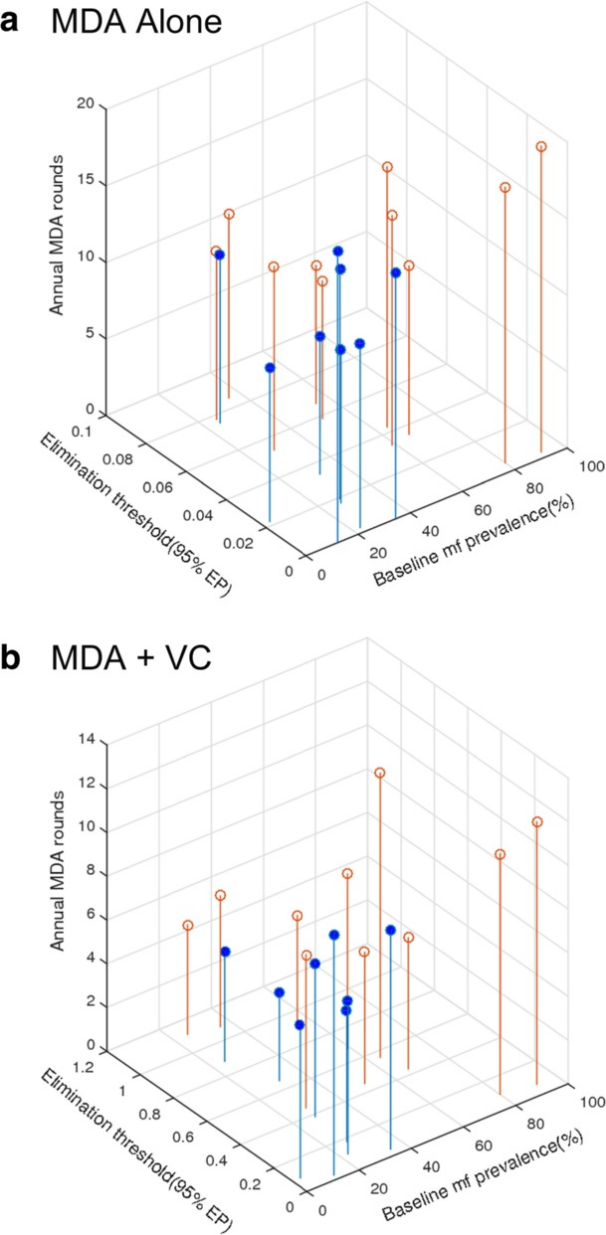
Mean rounds of annual MDAs in years predicted for achieving LF elimination as a joint function of the community-level baseline mf prevalence and breakpoint thresholds at 95 % EP. (**a**) MDA alone and (**b**) MDA + VC. Blue symbols, culicine sites; tan symbols, anopheline sites. EP, elimination probability; MDA, mass drug administration; VC, vector control

Figure 5 plots and compares the duration in years of annual MDA alone (at 80 % coverage) versus annual MDA plus vector (both administered at 80 % coverage) required to eliminate LF in relation to both the mf breakpoint value (at the 95 % EP) and the baseline mf prevalence prevailing in the current anopheline and culicine study sites. The results indicate that the duration of interventions needed to break LF transmission in a site is a complex outcome of both the elimination threshold value and baseline infection prevalence, which may intriguingly also depend on the associated transmitting vector species. Thus, while at low-moderate locality baseline mf prevalence levels, striking between-site variation may occur in the needed durations of the two LF interventions investigated here for achieving parasite elimination, as baseline mf prevalence increases in a site the durations of these interventions will increase significantly. However, this outcome appears less well demonstrated for the culicine compared to the anopheline sites investigated in this study (Fig. 5). While this may reflect an artefact of the smaller culicine study set used in this study, it is notable that culicines in general appear to be less efficient than anophelines in transmitting LF infection [39, 57], with lower levels of endemic mf prevalence produced at comparable community ABR values in culicine than in the case of anopheline settings (Table 1; [57]). This constraining of endemic infection prevalence could in turn restrict the range of breakpoint values in culicine settings leading to a lower range in the durations of interventions estimated for our culicine study sites compared to those obtained for anopheline sites. On the other hand, the higher endemic infection prevalences produced in the anopheline sites as ABR increases combined with the declining mf breakpoints at higher ABR values (Fig. 3) would increase the intensity and durations of interventions required to eliminate LF from such settings.

**Fig. 6:**
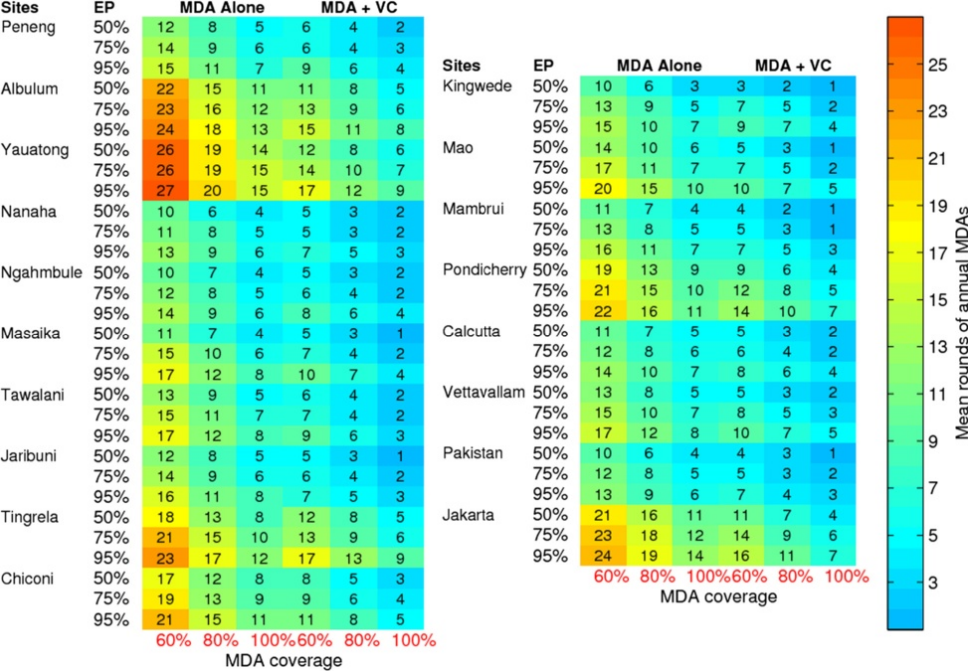
Mean rounds of annual MDAs in years for achieving LF elimination in each study site. The left and right heat maps are, respectively, for the anopheline and culicine settings. Two intervention scenarios (namely, MDA alone and MDA + VC, with VC coverage at 80 %) were modeled using three mf breakpoint threshold values at 50 %, 75 % and 95 % elimination probabilities (see Table 3). The results are shown for three MDA coverages at 60 %, 80 % and 100 % for the MDA alone in the first three columns and for the MDA + VC strategy in the remaining three columns of both the left- and right-panel plots. The drug regimens and their respective efficacies (i.e. adult worm and mf killing rates and efficacious period) used in modeling these intervention scenarios are given in Table 1. The mean number of years of interventions were derived using model runs for each of the 18 study sites based on their site-specific best-fit parameter vectors. EP, elimination probability; MDA, mass drug administration; VC, vector control

Figure 6 tabulates these outcomes for all study sites, and highlights the two major impacts on LF interventions arising from variations in intervention coverage and choice of EP threshold targets: 1) that durations of LF interventions for achieving transmission elimination in either vector setting and for each type of intervention will decrease with increasing intervention coverage; and 2) that they will increase significantly with the use of breakpoints signifying higher elimination probabilities. The latter finding illustrates the management trade-offs connected with the choice of EPs; i.e. that choosing a higher level of confidence for ensuring the meeting of transmission interruption or elimination (e.g. choosing a breakpoint value signifying a 95 % probability of elimination) will invariably lead to the need for implementing longer durations (and hence higher cost) of control regardless of MDA coverage and whether VC is included or not, compared to choosing a threshold with lower EP (say, 50 %). However, an important finding is that including VC will, by reducing the duration of interventions needed, drastically lower this cost of switching from using a lower EP to a robustly higher EP in all the current study settings (Fig. 6).

### Macroscopic predictions

**Fig. 7:**
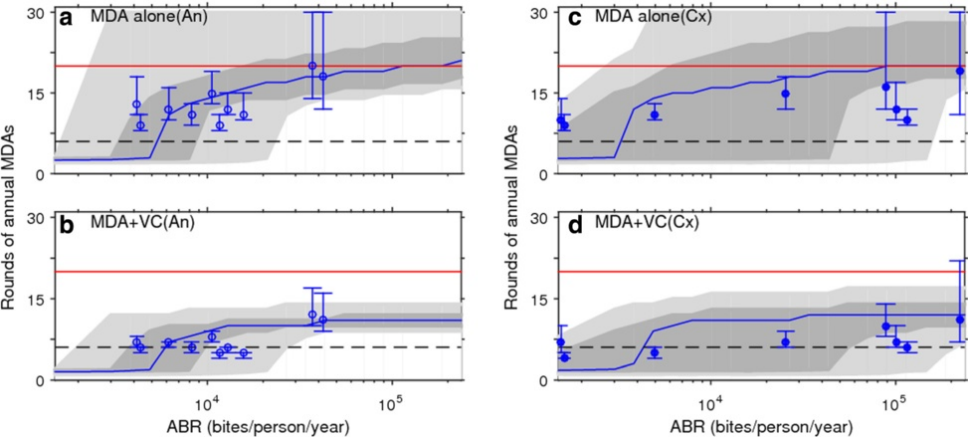
Site-specific versus macroscopic superensemble predictions of the impact of LF interventions. The results from combining site-specific best-fit model parameters to develop and use vector-specific superensemble models for simulating the impact of LF intervention at 80 % MDA and VC coverages for the MDA alone and MDA + VC strategies are shown in (**a**, **c**) and (**b**, **d**), respectively. The solid curves represent the superensemble medians of annual MDA rounds required to reduce community-level mf prevalences below their respective infection breakpoint thresholds for achieving a 95 % probability of elimination, and are stratified as a function of community ABR (annual biting rate) values. Note that the x-axis is on a logarithmic scale. The dark and light grey regions, respectively, represent the 50 % (between the 25th and 75th percentiles) and 95 % (between the 2.5th and 97.5th percentiles) credible intervals (CIs) of the number of years of interventions predicted by the ensemble model to cross the respective 95 % elimination thresholds in each site. Circles (open, anopheline sites; filled, culicine sites) denote the median number of years of each intervention (at 80 % coverages) predicted by the respective best-fitting site-specific models to break LF transmission. The lower dashed line drawn at 6 years (i.e. the time period representing six annual MDA rounds) is to contrast the model-predicted MDA rounds required to achieve LF elimination with the WHO recommendation of applying six annual MDAs to achieve elimination of LF from all endemic settings in the world. The upper solid line drawn at 20 annual MDA cycles represents the target deadline for meeting the call for eliminating LF worldwide by 2020. The results for each site represent simulations of the impact of interventions mimicking a start year of 2000 (i.e. the year of WHO announcement of GPELF) and maintenance of MDA and VC coverages at 80 % throughout

**Fig. 8:**
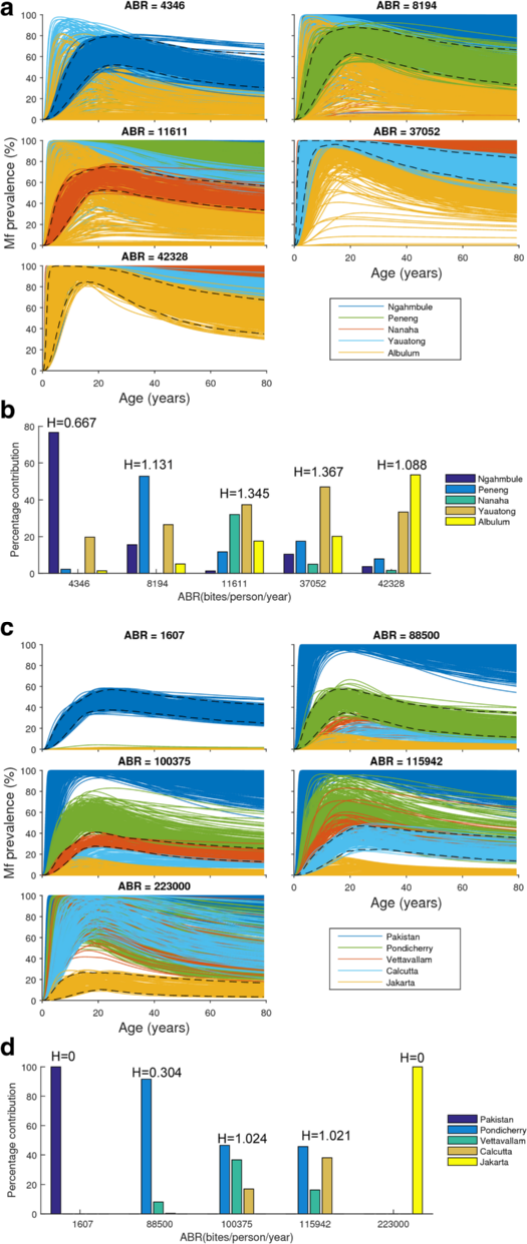
Contribution of site-specific parameter vectors to predictions of the superensemble model. The simulation of mf age-prevalence curves at endemic equilibrium by the vector-specific LF regional superensemble model (see text) given the baseline ABR of each study site are portrayed for each of five PNG anopheline (**a**, **b**) and five Southeast Asian culicine (**c**, **d**) study settings. The curves represent the sets of mf age-prevalence curves, individually color-coded, generated by the resultant *S* (=5) site-specific parameter vectors comprising the respective regional model in each site. In each site, we count the number *n*_*i*_ of the best-fit parameter vectors (belonging to the *i*th site-specific set of the superensemble) that are able to reproduce the observed mf age-prevalence in each site (i.e. fall within the 2.5th and 97.5th percentiles (shown by the dashed curves) of the site-specific mf age-prevalence data), in order to quantify the proportional contributions (i.e. $\frac{n_i}{N}$ where *N* = ∑*n*_*i*_) of individual members, *S*, of the global model to each site-specific prediction. The Shannon index, $H=-{\displaystyle {\sum}_{i=1}^S\left(\frac{n_i}{N} \ln \frac{n_i}{N}\right)}$ was used to measure the diversity in the superensemble parameter vectors as a result of the relative contributions of these *S* members to each regional prediction, with a higher diversity index denoting a greater contribution of site-specific parameter vectors arising from different study settings to the regional prediction of infection in a site. The bars in the grouped-bar plots in (**b**, **d**) depict the percentage contribution (i.e. $\frac{n_i}{N}\times 100$ of each *S* site-specific parameter member to the regional ensemble model predictions of age-infection in each of the anopheline (**b**) and culicine (**d**) settings, with the values of the corresponding Shannon index (H) displayed overhead

The results of intervention predictions for each superensemble model are given in Fig. 7. These highlight, firstly, that a macroscopic vector-specific LF ensemble model comprising of best-fit parameter vectors from all relevant sites is able to capture and hence adequately predict the number of years of MDA required to achieve local LF elimination as a function of ABR. However, the results indicate that there is a major trade-off with this global ability as it comes with a cost in the variability of making the macroscopic predictions that varies dramatically between the two interventions. Thus, while the predictions are highly variable in the case of the MDA alone intervention (Fig. 7a and c), this variability is drastically reduced in the MDA plus vector control case (Fig. 7b and d). The superensemble predictions are interestingly also comparatively less variable, particularly for the combined intervention strategy in the case of the anopheline system compared to the culicine case (Fig. 7). Figure 8 compares the contributions of the site-specific parameter vectors within the global superensemble model to the parameter vectors that best describe the mf age-prevalence curves observed given local ABR values in each of our study sites from either the anopheline (Fig. 8a and b) or culicine (Fig. 8c and d) settings. The dashed lines in each plot represent the 95 % upper and lower confidence band of the mf age-prevalence curve in each site, while the solid lines denote predictions of the site-specific parameter vectors making up the anopheline and culicine LF superensemble models—colored according to locality (Fig. 8)—in each of these sites. The relative contributions of the site-specific parameter vectors comprising a superensemble to the ensemble model fit to each dataset from a site can be discerned and calculated from the proportion of mf age curves predicted using the site-specific parameter vectors that fall within the mf curve band within each site. This can be seen both from the overlapping of curves predicted from the site-specific vectors of the superensemble model to a site’s observed age-prevalence curve (Fig. 8a and c), as well as the summary bar charts (Fig. 8b and d) below the age-pattern plots that show the calculated percentages of site-specific vectors from the superensemble that contributed to observed age-infection data in each site. The *H* values given above each bar group depict values of the Shannon index obtained by assessing the diversity of site-specific parameter vectors contributing to the superensemble predictions for a site. These formally indicate that site-specific parameters may play a greater role in superensemble model fits and hence ability to predict local infection dynamics in the case of anopheline compared to culicine filariasis (i.e. that anopheline transmission dynamics is comparatively less constrained by local ABR initial conditions). This comparative lesser local parameter constraining could consequently also underlie the lower variance observed in the superensemble predictions for this system (Fig. 7). However, despite the above results, for both vector systems, it is clear that using annual MDA alone will not allow meeting the goal of LF elimination using just the 6 years of annual treatment recommended by the WHO; in fact in sites with higher values of ABR, it will take up to >20 years (and dramatically beyond the year 2020 end date) to achieve this goal (Fig. 7a and c). Including vector control to MDA, however, will not only drastically reduce the number of annual MDAs, but for sites up to a moderate ABR value, it will also meet the goal of achieving LF elimination by just six rounds of treatment (Fig. 7b and d).

### Impact of ABR on transmission and extinction dynamics

**Fig. 9:**
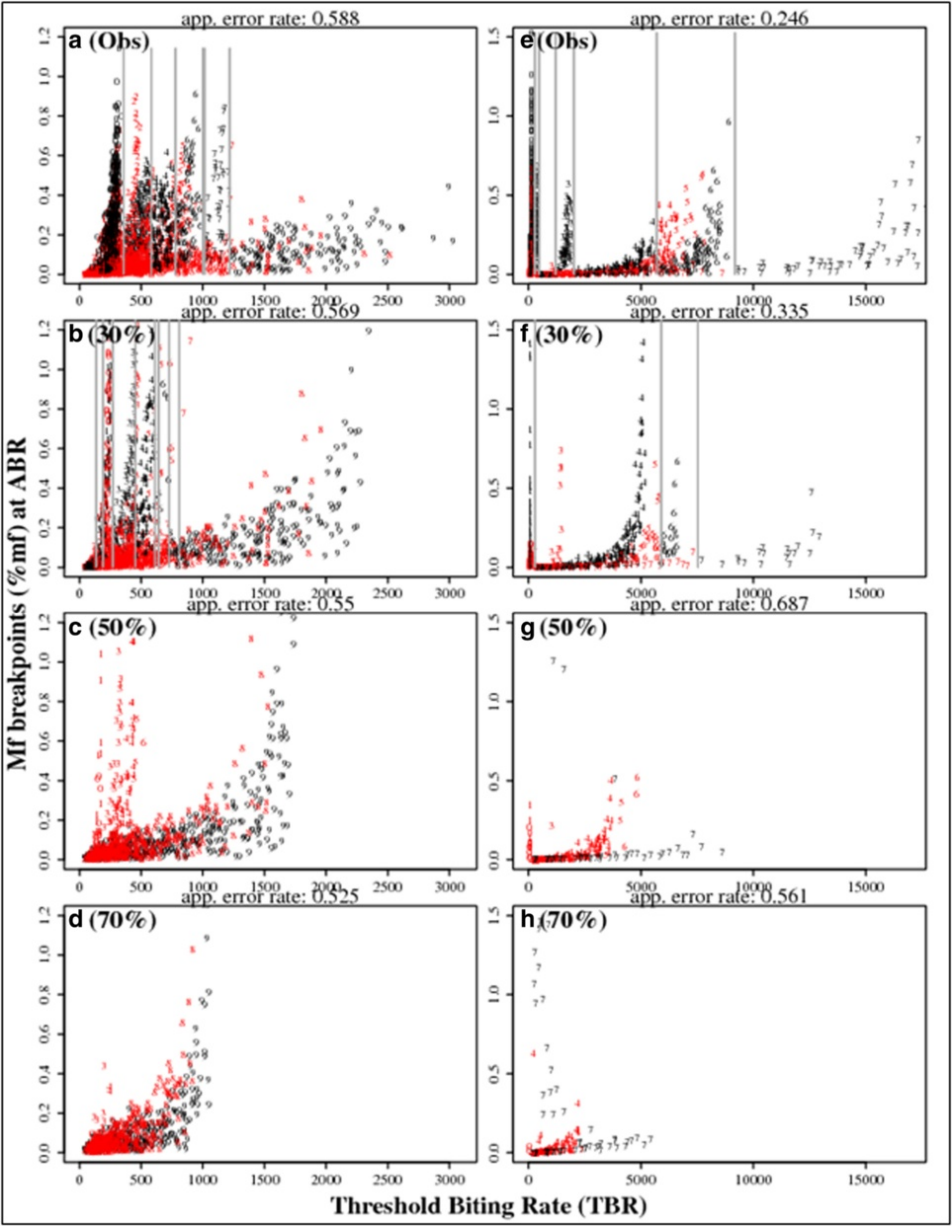
The impact of reducing ABR by VC on LF transmission regimes. The recursive partitioning of LF elimination regimes was obtained by carrying out a classification analysis using the kalR package in R on mf breakpoint values obtained at different ABR values changing from baseline due to reductions brought about by VC. The left-side panel of plots (**a** to **d**) portray the results for the anopheline (*An*) superensemble whereas the right-side panel (**e** to **h**) show results for the culicine (*Cx*) global model. Mf breakpoints depicted in each panel plot were calculated at the observed baseline ABR values (**a**(Obs) and **e**(Obs)) and at reduced ABR values per site as follows: 30 % reduction (**b**, **f**); 50 % (**c**, **g**); and 70 % (**d**, **h**). As the baseline ABR values in each site are reduced from 0 % (no reduction) to 30 %, different regimes of breakpoints signifying initially separable or partitionable site-specific values as indicated by the vertical lines begin to shrink in terms of their ranges. Further reductions (of 50 % and 70 %) in the baseline ABRs lead to a collapse of these different regimes into a single regime at the 70 % reduction stage

Figure 9 shows results from a recursive partitioning analysis [58] of temporal changes in individual site-specific mf % breakpoints from baseline to a sequence of states when ABR is progressively reduced cumulatively over time by VC. The results underline a major outcome arising from the use of VC that may underlie the reduction in the variability of the MDA plus vector control predictions depicted in Fig. 7, namely that this could primarily be due to a dissolution in the between-study heterogeneity in these breakpoints brought about as a result of VC-induced negative changes in the prevailing abundance of vectors. Indeed, the results show that (for both LF-vector combinations) at high (50 % and 70 %) levels of ABR reductions, initially separable between-site breakpoint values converge until there is effectively only a single regime of unpartitionable breakpoints that remain among the still infection-positive sites. This finding supports our previous conclusion [11] that ABR may represent the major factor bounding the local transmission and extinction dynamics of LF, and that including VC could effectively compress such widely differing ABR-driven locality-specific LF transmission regimes (here as measured by site-specific mf breakpoint values) into a single regime if it can be applied at levels that can lead to consistently large declines in the prevailing vector populations.

## Discussion

The chief contributions of this modelling study of the dynamics of LF elimination based on detailed parasitological and entomological field data are twofold. First, we have advanced knowledge regarding the nature and the organizational features that underlie heterogeneous LF transmission across endemic localities, and the effects these have for infection and vector-related elimination thresholds. The key result here most immediately relevant to global LF elimination is the finding that, as a result of parameter adjustment to local transmission environments, significant differences in parasite population dynamics and in the resultant transmission and infection breakpoints occurred between the 18 endemic villages investigated. Further, given our Monte Carlo ensemble-based data-modelling framework that was designed to capture local uncertainty and variability in transmission parameters from site-specific data [8, 11, 31, 44, 59], we show that rather than being a single estimate, both these infection-related and vector abundance thresholds can exist as a “cloud” or distribution of values within and between village sites, with each value related to a probability that parasite elimination will be achieved when crossed [56]. This has significant strategic implications as it clarifies that there is a choice in choosing a threshold value from such distributions to serve as an endpoint or breakpoint target in management programs, and as can be seen from Table 3, given that these threshold values can range from as high as 3 % mf prevalence (for worm or infection breakpoints) to as low as 0.0002 %, such a choice ultimately revolves on how risk of program failure is (implicitly or explicitly) perceived and accepted by the relevant policy makers; i.e. whether management or the decision maker is risk averse (and hence opts for high confidence (e.g. 95 % probability) of achieving elimination) or risk tolerant (and so is tolerant of using values signifying lower confidences of achieving elimination). It is instructive to note, in this regard, that the WHO currently promotes the use of a 1 % mf prevalence threshold to serve as the elimination target for MDA programs globally [60]; our results on mf prevalence breakpoint values (Table 3) indicate that such a target is likely to afford at best only a moderate level of confidence (up to at best 80 % probability of elimination) that LF transmission will be interrupted when this value is used globally or invariantly as a metric to signify program success.

The present work has provided intriguing new insights concerning the factors that may underlie LF transmission adaptation and response to both local environmental conditions and intervention-induced perturbations. An important finding is that local transmission adaptation appears to be governed by only a few biological parameters, with the majority of these parameters poorly constrained by local data. This feature, previously primarily thought of as being an outcome of either poor or lack of parameter identifiability [33, 61], has recently been shown instead to be an intrinsic feature of complex multiparameter biological systems [34, 53, 62]; i.e. that often it is not possible to identify or estimate values for many parameters of these systems even with the availability of detailed data [63]. This phenomenon, which has been termed as “parameter sloppiness”, is attributed to the existence of a highly anisotropic structure in the parameter space, wherein the behaviour of these systems is insensitive to perturbations in the majority of its defining parameters while varying due to changes on only a few “stiff” combinations of model parameters [34, 62]. Our results in this study indicate that this system characteristic may also apply to the transmission dynamics of parasitic infections; however, they also highlight that while such “sloppy” parameter behaviour has the potential to make global LF transmission invariant or robust to many local permutations or changes in environmental conditions, including as we have shown previously to temporally varying follow-up infection data in response to interventions in a setting [11], this sloppiness may have evolved at the local level to withstand variations across a relatively narrow range or thresholds of environmental shocks (i.e. the LF system may be robust to changes in initial conditions within only a set of local constraint values [64]), with the local system commensurately susceptible or fragile to shocks outside these thresholds (but see below).

This behaviour of the LF system, particularly the robust (i.e. maintenance of transmission despite external and internal perturbations [32]) yet fragile (extreme sensitivity leading to transmission disruption following perturbations) duality of transmission/extinction dynamics in relation to environmental variability in vector abundance, suggests that LF transmission may be an example of a highly optimized tolerance (HOT) system [65–67], the structure and operation of which have been the basis of new lines of enquiry and thinking regarding mechanisms that may govern the robustness and persistence of complex systems [32, 68–70]. Such work on HOT architectures across various biological systems has shown that a key mechanism that generates robustness is increasing complexity in the internal structure of a system, wherein many variables and feedback loops have been tuned to favor or accommodate small losses in system function/productivity in response to common events at the expense of large losses when subject to unexpected perturbations [66–68]. We show in Fig. 3 the likely operation of this mechanism in the case of LF transmission, whereby decreases in worm breakpoint values as a function of mosquito abundance follow power-law functions, rather than the comparatively faster decreases that would be expected if exponential relationships were to occur between these states [71]. This result implies that the cost of maintaining the complex internal structure required to accommodate common disturbances in the LF system is the occurrence of relatively high worm breakpoint values; it also suggests that ABR values in a locality may govern the structural configuration of LF transmission to local conditions, and that inducing changes in ABR values outside the normal range experienced locally would provide an effective mechanism to significantly increase transmission fragility, and hence affect reliable disruption of infection.

The assessments carried out in the second half of this study in relation to evaluating the impact that site-specific heterogeneity in transmission dynamics may have on the prospects for eliminating LF has provided important first insights as to how such mechanisms operate and may impact current options to interrupt LF transmission. Our chief finding in this regard is that this interplay between LF transmission organization and dynamics at the local level will significantly influence the durations of control required to break parasite transmission in a setting. We show specifically that control durations will vary from site to site as a result of complex interactions between local transmission intensity, efficiency, breakpoints, and robustness to environmental changes or perturbations, but also with respect to the type of interventions being applied as well as the transmitting vector genus. Thus, we found that while durations of interventions will significantly vary between our study sites, these durations will generally be longer and much more variable when using the MDA alone strategy (with years of interventions varying between 6 and 20 years at 80 % drug coverage) compared to the MDA plus vector control strategy (with the years of interventions ranging between 2 and 13 at the same 80 % drug and vector control coverages (Fig. 6)). As we show in Fig. 9, this difference between the two interventions is largely a function of the transmission regime homogenization or convergence brought about by vector control, which by reducing the robustness of LF transmission to change in the local dynamics constraining variables and facilitating the switching of transmission dynamics into a more narrowed and more fragile regime (in terms of increasing infection breakpoint values), can lead to a decrease in the extent and variance in the intervention durations required to disrupt parasite transmission. By contrast, the results imply that the higher variability and longer durations of interventions required when applying the annual MDA strategy alone are likely to be a function of the strong density-dependent negative feedback loops, such as those fostered by the limitation, acquired immunity and worm mating functions [7, 72], that govern LF transmission in endemic areas compensating variably for the worm killing effects of drug treatments. These findings clearly indicate that gaining a better understanding of the interactions between system structures that generate robustness and the specific perturbations being applied to a system will be crucial to identifying the informed locally adaptive strategies required for achieving the reliable disruption of parasite transmission from all endemic settings [70]. From this perspective, it is clear that reducing vector abundance in addition to killing worms using MDA, by significantly increasing the fragility of transmission, may be a better option than applying MDA alone for effectively eliminating LF transmission.

Another significant and unexpected, but intriguing finding from the intervention simulations carried out here relates to the fact that despite the lower estimates of infection breakpoints in the culicine study sites, the durations of interventions for these sites, irrespective of type, are calculated to be within the range predicted for the anopheline settings for similar low to medium pre-control community vector biting rates; i.e. between 5 to 15 years in general (Fig. 5). Given that the generally lower mf breakpoint values estimated for the culicine study sites (Table 3) would have indicated the need for longer durations of interventions in these sites in comparison with the anopheline case, this finding thus suggests that factors other than breakpoint values may also play a role in governing the LF system response to interventions. Our results show that one factor underlying this paradox may relate to the robustness-performance trade-offs that govern the two LF systems. Thus, we show firstly that although transmission breakpoints are lower in the case of culicine LF, the performance or production efficiency of this system in terms of the overall mf prevalence produced for the same ABR is lower than that of the anopheline system [57]. This would result in a smaller distance or basins of attraction between endemic infection levels relative to elimination thresholds in the culicine compared to the anopheline system [7], an outcome that could clearly overcome the impact that lower breakpoint values estimated for this system (Table 3) may have on lengthening intervention durations. Note that as different assemblages of density-dependent mechanisms govern the differential levels of infection and breakpoints values generated in each system [7], wherein in one case (culicine), strong negative density-dependent factors, such as the L3 limitation function and host acquired immunity, lowers the endemic mf levels reached but also slows the approach to crossing the lower extinction thresholds (hence enhancing the stability of the endemic state) and in the anopheline case, strong positive density-dependent functions, such as the L3 facilitation and host immunosuppression functions, lead to higher endemic mf prevalences but faster approaches to higher extinction thresholds over the same ABR ranges, our finding of a strong vector specificity in the response of the parasite population to different LF control interventions further supports our overall contention from this study that it is the complex interplay between dynamics and the internal organization structure underlying LF transmission—in terms of resource use, productivity and robustness—that will ultimately underlie the dynamics of LF elimination in an endemic setting [32, 68, 70, 73, 74].

The evaluations carried out in this study with regards to examining the feasibility of developing and using superensemble models of LF transmission, based on pooling site-specific parameter vectors, to facilitate predictions of the impact of interventions at the macroscopic scale was predicated on the hypothesis that sloppiness in parameter values would indicate a weak dependence on microscopic details and thus allow effective macroscopic predictions. It was also based on growing work on multiparameter models from a range of fields, including physics and biology, that has underscored how such sloppiness in parameter values may be the key factor underlying the ability of mathematical models in predicting complex phenomena at larger scales despite considerable microscopic uncertainty [34, 62]. We show here for the first time that indeed such macroscopic superensemble models would be able to predict the number of years of LF interventions required to achieve LF elimination in different sites varying in baseline mf prevalence and ABR values. However, a major finding is that the ability of these global models to make reliable predictions is critically dependent on both the type of LF interventions being modelled and on the vector species mediating transmission in a locality (Fig. 7). Thus, while the results indicate how comparatively more reliable (lower variance) predictions of the effects of combined MDA and vector control are possible owing to the pushing of the LF system into common dynamical regimes as a result of ABR reductions (discussed above), an unexpected finding was that intervention predictions using the constructed superensemble models were also more reliable for anopheline compared to culicine LF. We suggest that this is largely due to the greater constraining of culicine dynamics to local settings; i.e. culicine model parameters may be relatively less sloppy than in the case of the anopheline parameters (Fig. 8). This implies that the robustness of the culicine system may be restricted to changes of initial conditions within a fixed local boundary of ABR values, whereas anopheline LF could also be robust to changes in these constraining values between sites. This difference in the type of robustness clearly makes it possible to undertake a more reliable macroscopic modelling of anopheline LF transmission dynamics and control using the present superensemble modelling approach, and highlights how apart from affecting the outcomes of interventions, biological organizational architectures that govern transmission robustness may also govern the practical ability of models to make reliable macroscopic predictions of the effect of specific interventions. However, note a trade-off is that such robustness may also reduce the capacity of anopheline LF for evolutionary and environmental adaptation relative to culicine LF [33, 70]. This is an important finding because if times to genetic rescue become favourable in relation to those that would bring about population extinction as a result of LF interventions [36], then we predict that culicine systems would be more likely to evolve drug resistance, say, as a specific example of a mutational response to MDA, compared to anopheline LF. The practical conclusion of this finding is clear, namely that if drug resistance to LF MDA emerges this will occur first in culicine areas and thus that management options, for example combined MDA plus vector control [36, 75], to prevent such an eventuality, as well as surveillance for detecting mutational changes reflective of developing resistance, should also be targeted in the first instance to these areas.

## Conclusions

We have shown in this study for the first time how the multiple aspects that characterize biological robustness to a set of perturbations and its expression in terms of system resource demands, productivity and structure, will not only lead to a better understanding of heterogeneous LF transmission dynamics and persistence but also to delineating and identifying the set of external conditions and perturbations that would reliably increase system fragility and hence lead to a more predictable disruption of LF transmission. This is an important result and indicates how understanding the complex ecology of parasite transmission and persistence, rather than merely basing decisions on empirical field or clinical trial results, is central to the development of effective control or elimination strategies. We show in this regard, for example, how including vector control to MDA may not only reliably increase system fragility and hence reduce the number of years of interventions required to interrupt LF transmission significantly—in many cases to within the WHO recommended 6 years of intervention—but by also additionally reducing transmission regime variability permit the making of more reliable global predictions of control requirements. These findings imply that a change in thinking is now required concerning how parasite elimination programs are to be designed if we are to identify and apply better approaches to disrupting transmission. More specifically, they suggest that the use of robustness, including features of HOT mechanisms, as a design principle to investigate the nature of, and response to, assemblages of intervention options, could provide a more effective framework and tool for uncovering options that would reliably and sustainably eliminate LF, and indeed other parasitic diseases, from all settings in the face of extant environmental heterogeneity and uncertainty, and possibly even problems previously unencountered (e.g. evolution of drug resistance by LF parasites). We suggest that adaptive modelling methods, such as the coupled data-modelling approach developed here, that will allow the construction of robustness profiles of parasitic systems in response to environmental variations may provide a first step in this process [74, 76, 77]. We also echo in this regard increasing calls for the assembly and release of LF intervention data from the many countries collecting these data as part of their LF program monitoring and evaluation activities to modellers so that predictions made in the present study could be verified and tested rigorously. Given the current pressing policy needs of the global LF elimination program, and indeed other growing neglected tropical disease control programs, we indicate that this work be urgently initiated in order that the goal of eliminating these major diseases of the global poor is more robustly supported.

## Additional file

Additional file 1: **Supplementary Material.** (DOCX 1731 kb)

## Abbreviations

ABR: annual biting rate
ALB: albendazole
BM: Bayesian melding
CDF: cumulative density function
DA: data-model assimilation
DEC: diethylcarbamazine citrate
EP: elimination probability
GPELF: global programme to eliminate lymphatic filariasis
HOT: highly optimized tolerance
IRS: indoor residual spray
IVM: ivermectin
LF: lymphatic filariasis
LLIN: long-lasting insecticidal net
MBR: monthly biting rate
MDA: mass drug administration
mf: microfilariae
SIR: sample importance resampling
TBR: threshold biting rate
VC: vector control
WHO: World Health Organization
