# Supplementary material for: Heterogeneous dynamics, robustness/fragility trade-offs, and the eradication of the macroparasitic disease, lymphatic filariasis
Source: BMC Med. 2016 Jan 28;14:14. doi: 10.1186/s12916-016-0557-y (PMC4731922; doi:10.1186/s12916-016-0557-y)
Supplement: Supplementary file 1 — Supplementary Material. (DOCX 1731 kb) [file 12916_2016_557_MOESM1_ESM.docx]

Additional File 1

**Additional Table S1** - **Description the basic LF model parameters and functions used in the model.**

| **Parameter**  **Symbol** | **Definition**  **(*units*)** | **Range** | **Source** |
| --- | --- | --- | --- |
| Intrinsic ***Biological*** parameters | | | |
| ***λ*** | Number of bites per mosquito (*per month*) | [5, 15] | [1-5] |
| ***ψ_1_*** | Proportion of L3 leaving mosquito per bite | [0.12, 0.7] | [6] |
| ***ψ_2_*** | The establishment rate^1^ | [0.0000398, 0.00364] | [3-5, 7] |
| ***μ*** | The worm mortality rate  (*per month*) | [0.008, 0.018] | [3-5, 8-11] |
| ***α*** | Production rate of microfilariae per worm  (*per month*) | [0.25, 1.5] | [3-6] |
| ***γ*** | The death rate of the microfilariae  (*per month*) | [0.08, 0.12] | [4-6, 10] |
| ***g*** | Proportion of mosquitoes which pick up infection when biting an infected host | [0.259, 0.481] | [4, 5, 12] |
| ***σ*** | Death rate of mosquitoes  (*per month*) | [1.5, 8.5] | [4, 5, 7] |
| ***κ*** | Maximum level of L3 given mf density | [3.955, 4.83] | [4, 5] |
| ***c*** | Strength of acquired immunity | [0.0000003, 0.0109] | [4, 5] |
| ***$\delta$*** | Immunity waning rate (*per month*) | [0, 0.000001] | [4, 5] |
| Extrinsic ***Biological*** parameters | | | |
| ***V/H*** | Ratio of number of vector to hosts | $MBR/\lambda$ | Data (Table 1) |
| ***H_Lin_*** | A threshold value used in$h(a)$to adjust the rate at which individuals of age *a* are bitten: linear rise from 0 at age zero to 1 at age *H_lin_* in years.$h(a)=a/H_{Lin}\text{ for }a<H_{Lin};h(a)=1\text{ for }a\geq H_{Lin}$ | [12, 240] months | [4, 5, 13] |
| ***r*** | Gradient of mf uptake^2^ | [0.0495, 0.22] | [4, 5] |
| ***I_C_*** | Strength of immunosuppression^3^ | [0.5, 5.5] | [4, 5] |
| ***S_C_*** | Slope of immunosuppression function^4^  (*per worm/month*) | [0.01, 0.19] | [4, 5] |
| ***k_0_*** | The basic location parameter of negative binomial distribution used in aggregation parameter  ($k=k_{0}+k_{Lin}M$) | [0.000036, 0.00077] | [4, 5, 14, 15] |
| ***k_Lin_*** | The linear rate of increase in the aggregation parameter defined above | [0.00000024, 0.282] | [4, 5, 14, 15] |
| ***Description of the functions used in the model*** | | | |
| **Function** | **Mathematical expression** | **Parameters** | **Source** |
| Probability that an individual is of age *a*  *π(a)* | $\pi(a)=0.0361\exp[-0.0304a]$ | Human age *a* in month | [4, 5, 13] |
| Adult worm mating probability *ϕ(W,k)* | $1-\left( 1+\frac{W}{2k} \right)^{-\left( 1+k \right)}$ | *k* – negative binomial aggregation parameter | [3-5, 16] |
| Immunity to larval establishment *g_1_(I)* | $\frac{1}{1+cI}$ | *c* – strength of immunity to larval establishment | [4, 5] |
| Host immune-suppression  *g_2_(W)* | $\frac{1+I_{C}S_{C}W}{1+S_{C}W}$ | *I_C_* – strength of immunosuppression;  *S_C_* – slope of immunosuppression | [4, 5] |

^1^The proportion of L3-stage larvae infecting human hosts that survive to develop into adult worms [4].

^2^The gradient of mf uptake *r* is a measure of the initial increase in the infective L3 larvae uptake by vector as *M* increases from 0 [4, 13].

^3^ The facilitated establishment rate of adult worms due to parasite-induced immunosuppression in a heavily infected human host

^4^ The initial rate of increase by which the strength of immunosuppression is achieved as *W* increases from 0 [17].

Additional File 1

**Additional Table S2** - **Monte Carlo *p*-values (***age-stratified* **and** *overall***)** [18] **for the fitted models to the baseline infection data collected all the 18 study sites.**

| **Peneng** | **0 to 10** | **10 to 20** | **20 to 30** | **30 to 40** | **40 to 50** | **50 to 60** | **Overall** |  |  |  |  |  |  |  |
| --- | --- | --- | --- | --- | --- | --- | --- | --- | --- | --- | --- | --- | --- | --- |
|  | 0.749 | 0.348 | 0.966 | 0.865 | 0.999 | 0.87 | 0.998 |  |  |  |  |  |  |  |
| **Albulum** | **0 to 10** | **10 to 20** | **20 to 30** | **30 to 40** | **40 to 50** | **55 to 65** | **Overall** |  |  |  |  |  |  |  |
|  | 0.779 | 0.972 | 0.311 | 0.85 | 0.985 | 0.999 | 0.998 |  |  |  |  |  |  |  |
| **Yauatong** | **0 to 10** | **10 to 20** | **20 to 30** | **30 to 40** | **40 to 50** | **50 to 60** | **60 to 70** | **Overall** |  |  |  |  |  |  |
|  | 0.769 | 0.997 | 0.831 | 0.987 | 0.997 | 0.999 | 0.051 | 0.999 |  |  |  |  |  |  |
| **Nanaha** | **0 to 10** | **10 to 20** | **20 to 30** | **30 to 40** | **40 to 50** | **50 to 60** | **60 to 70** | **Overall** |  |  |  |  |  |  |
|  | 0.999 | 0.945 | 0.827 | 0.622 | 0.457 | 0.999 | 0.999 | 0.999 |  |  |  |  |  |  |
| **Ngahmbule** | **0 to 10** | **10 to 20** | **20 to 30** | **30 to 40** | **40 to 50** | **50 to 60** | **60 to 70** | **Overall** |  |  |  |  |  |  |
|  | 0.736 | 0.865 | 0.715 | 0.78 | 0.764 | 0.999 | 0.984 | 0.972 |  |  |  |  |  |  |
| **Masaika** | **1 to 4** | **5 to 9** | **10 to 14** | **15 to 19** | **20 to 29** | **30 to 39** | **40 to 49** | **50 to 59** | **60 to 69** | **Overall** |  |  |  |  |
|  | 0.989 | 0.678 | 0.156 | 0.678 | 0.567 | 0.999 | 0.889 | 0.999 | 0.999 | 0.999 |  |  |  |  |
| **Tawalani** | **0 to 4** | **6 to 9** | **11 to 14** | **16 to 19** | **20 to 29** | **30 to 39** | **40 to 49** | **50 to 59** | **60 to 69** | **Overall** |  |  |  |  |
|  | 0.999 | 0.36 | 0.904 | 0.999 | 0.999 | 0.999 | 0.735 | 0.794 | 0.294 | 0.999 |  |  |  |  |
| **Jaribuni** | **1 to 4** | **5 to 9** | **10 to 14** | **15 to 19** | **20 to 29** | **30 to 39** | **40 to 49** | **50 to 59** | **60 to 69** | **70 to 79** | **Overall** |  |  |  |
|  | 0.999 | 0.886 | 0.835 | 0.999 | 0.608 | 0.304 | 0.823 | 0.582 | 0.999 | 0.999 | 0.999 |  |  |  |
| **Tingrela** | **0 to 5** | **6 to 10** | **11 to 15** | **16 to 20** | **21 to 30** | **31 to 40** | **41 to 50** | **51 to 60** | **Overall** |  |  |  |  |  |
|  | 0.771 | 0.19 | 0.82 | 0.998 | 0.618 | 0.362 | 0.9 | 0.999 | 0.97 |  |  |  |  |  |
| **Chiconi** | **10 to 19** | **20 to 29** | **30 to 39** | **40 to 49** | **50 to 59** | **60 to 69** | **Overall** |  |  |  |  |  |  |  |
|  | 0.206 | 0.984 | 0.997 | 0.763 | 0.981 | 0.999 | 0.999 |  |  |  |  |  |  |  |
| **Kingwede** | **1 to 4** | **5 to 9** | **10 to 14** | **15 to 19** | **20 to 29** | **30 to 39** | **40 to 49** | **50 to 59** | **60 to 69** | **Overall** |  |  |  |  |
|  | 0.97 | 0.991 | 0.991 | 0.78 | 0.152 | 0.999 | 0.982 | 0.982 | 0.999 | 0.997 |  |  |  |  |
| **Mao** | **0 to 4** | **6 to 9** | **11 to 14** | **16 to 19** | **20 to 29** | **30 to 39** | **40 to 49** | **50 to 59** | **60 to 69** | **Overall** |  |  |  |  |
|  | 0.989 | 0.78 | 0.484 | 0.989 | 0.999 | 0.857 | 0.846 | 0.714 | 0.999 | 0.999 |  |  |  |  |
| **Mambrui** | **1 to 4** | **5 to 9** | **10 to 14** | **15 to 19** | **20 to 29** | **30 to 39** | **40 to 49** | **50 to 59** | **60 to 69** | **70 to 79** | **Overall** |  |  |  |
|  | 0.91 | 0.36 | 0.16 | 0.71 | 0.999 | 0.999 | 0.999 | 0.5 | 0.83 | 0.999 | 0.999 |  |  |  |
| **Pondicherry** | **0 to 5** | **6 to 10** | **11 to 15** | **16 to 20** | **21 to 30** | **31 to 40** | **41 to 50** | **51 to 60** | **61 to 70** | **Overall** |  |  |  |  |
|  | 0.492 | 0.034 | 0.408 | 0.702 | 0.79 | 0.115 | 0.641 | 0.969 | 0.999 | 0.939 |  |  |  |  |
| **Calcutta** | **0 to 4** | **5 to 9** | **10 to 14** | **15 to 19** | **20 to 24** | **25 to 34** | **35 to 44** | **45 to 54** | **55 to 64** | **Overall** |  |  |  |  |
|  | 0.964 | 0.937 | 0.991 | 0.999 | 0.495 | 0.315 | 0.901 | 0.577 | 0.91 | 0.964 |  |  |  |  |
| **Vettavallam** | **1 to 4** | **5 to 9** | **10 to 14** | **15 to 19** | **20 to 29** | **30 to 39** | **40 to 49** | **50 to 59** | **60 to 69** | **Overall** |  |  |  |  |
|  | 0.999 | 0.094 | 0.283 | 0.396 | 0.849 | 0.83 | 0.075 | 0.981 | 0.981 | 0.999 |  |  |  |  |
| **Pakistan** | **1 to 4** | **6 to 9** | **11 to 14** | **16 to 19** | **20 to 24** | **25 to 29** | **30 to 34** | **35 to 39** | **40 to 44** | **45 to 49** | **50 to 54** | **55 to 59** | **60 to 64** | **Overall** |
|  | 0.953 | 0.482 | 0.995 | 0.016 | 0.987 | 0.045 | 0.945 | 0.733 | 0.963 | 0.911 | 0.882 | 0.999 | 0.999 | 0.999 |
| **Jakarta** | **0 to 4** | **5 to 9** | **10 to 14** | **15 to 19** | **20 to 24** | **25 to 29** | **30 to 34** | **35 to 39** | **40 to 44** | **45 to 49** | **50 to 54** | **Overall** |  |  |
|  | 0.906 | 0.413 | 0.964 | 0.174 | 0.362 | 0.246 | 0.999 | 0.232 | 0.957 | 0.999 | 0.21 | 0.92 |  |  |

Additional File 1

**Table S3 - Test results for differences in mf breakpoints, threshold biting rates (TBRs), and the baseline annual biting rates (ABRs) between study villages in the anopheline and culicine villages, as well as by mosquito species.** We used a Binomial Generalized Linear Model for testing between-villages/species differences in mf breakpoints, while a one-way ANOVA was used for differences in TBRs and a Wilcoxon signed rank test was used to test for between-village differences in baseline ABRs.

| **Test groups** (Mf Breakpoints) | **Df** | **Chi-squared** | ***p*-values** |
| --- | --- | --- | --- |
| **Anopheline sites** (10 in total) |  |  |  |
| Mf breakpoints (at TBR) by village | 9 | 1119.993 | < 0.0001 |
| Mf breakpoints (at ABR) by village | 9 | 1294.745 | < 0.0001 |
| **Culicine sites** (8 in total) |  |  |  |
| Mf breakpoints (at TBR) by village | 7 | 814.9113 | < 0.0001 |
| Mf breakpoints (at ABR) by village | 7 | 393.2089 | < 0.0001 |
| **By species (culicine versus anopheline*)*** |  |  |  |
| Mf breakpoints at ABRs by species | 1 | 0.1422 | 0.7061 |
| Mf breakpoints at TBRs by species | 1 | 113.598 | < 0.0001 |
|  |  |  |  |
| **Test groups** (TBRs) | **Dfs** (group, residuals) | **F-value** | ***p*-values** |
| **Anopheline sites** (10 in total) |  |  |  |
| TBRs by village | (9, 2445) | 127 | < 0.0001 |
| **Culicine sites** (8 in total) |  |  |  |
| TBRs by village | (7, 1449) | 1286 | < 0.0001 |
| **By species (culicine versus anopheline*)*** |  |  |  |
| TBRs by species | (1, 3910) | 7.119 | 0.00766 |
|  |  |  |  |
| **Test groups** (ABRs) | **V** |  | ***p*-values** |
| Anopheline ABRs | 55 |  | 0.001953 |
| Culicine ABRs | 36 |  | 0.007813 |
| All ABRs | 171 |  | < 0.0001 |

Additional File 1

**Table S4 - Results of one-way ANOVA for differences in the required MDA rounds between study villages in the anopheline and culicine settings, as well as by mosquito species.** The required MDA rounds were tested for the two strategies: MDA alone and MDA + VC. The results used here were from the simulated interventions at 80% coverages of MDA and VC (where applicable), at the elimination threshold of 95% EP.

| **Test groups** | **DFs** (group, residuals) | **F value** | ***p*-values** |
| --- | --- | --- | --- |
| **Anopheline sites** (10 in total) |  |  |  |
| Number of the required MDA rounds (MDA alone) | (9, 2445) | 831.9 | < 0.0001 |
| Number of the required MDA rounds (MDA + VC) | (9, 2445) | 2532 | < 0.0001 |
| **Culicine sites** (8 in total) |  |  |  |
| Number of the required MDA rounds (MDA alone) | (7, 1449) | 370.6 | < 0.0001 |
| Number of the required MDA rounds (MDA + VC) | (7, 1449) | 661.6 | < 0.0001 |
| **By species** |  |  |  |
| Number of the required MDA rounds in MDA Alone | (1, 3910) | 188.7 | < 0.0001 |
| Number of the required MDA rounds in MDA + VC | (1, 3910) | 217 | < 0.0001 |

Additional File 1

**
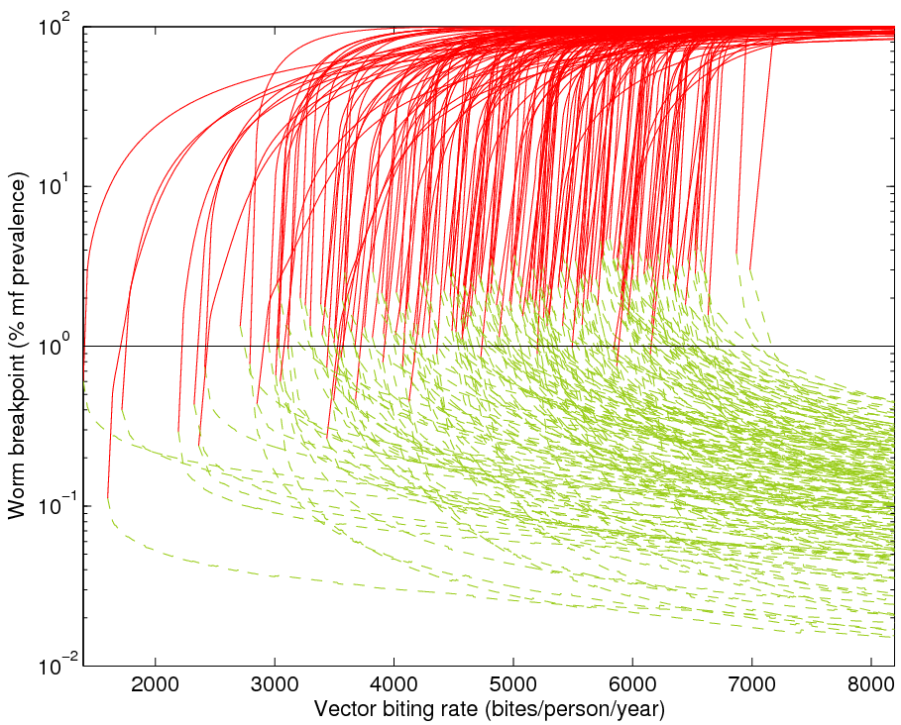
**

Additional Figure S1 - Numerical stability analysis of the fitted models to the baseline infection prevalence data. The results are shown for the SIR-selected parameter vectors which produced the best model fits to the Peneng baseline data shown in Figure 1. The upper (*red* solid lines) and lower branches (light *green* dashed line), respectively, represent the endemic stable and the unstable % overall mf prevalence - the latter prevalence values serving as a unstable boundary between the stable endemic infection and zero infection states, and thus representing the infection breakpoint thresholds above which the system moves to an endemic equilibrium and below which it moves to the zero attractor [3] - as a function of annual biting rate (ABR) of vector mosquitoes, labeled as *vector biting rate* on the x-axis. Note that the values of % mf prevalence on the y-axis are shown on the logarithmic scale. The solid horizontal line drawn at 1% mf prevalence is provided to guide the eye to take note of how the worm breakpoint value estimated at a prevailing baseline ABR (*e.g.* 8194 bites/person/year for the Peneng site) will steadily increase as ABR values decrease from right to left. The maximum worm breakpoint is reached at the threshold biting rate (TBR), the value of vector biting rate at which the two branches - the lower (unstable) and upper (stable) ones - meet. These two sets of worm breakpoint values, one set of values at the prevailing ABR and the other at TBR, were used to calculate the extinction threshold values for 50%, 75% and 95% probability of successful transmission interruption. As mass drug administration (MDA) does not change ABR values, infection breakpoints at ABR are relevant for modelling parasite elimination using MDA alone; by contrast, as vector control reduces ABR progressively towards the TBR values, the higher maximal infection breakpoints obtained at TBR are more relevant targets for infection elimination using the combined MDA plus VC intervention.

Additional File 1


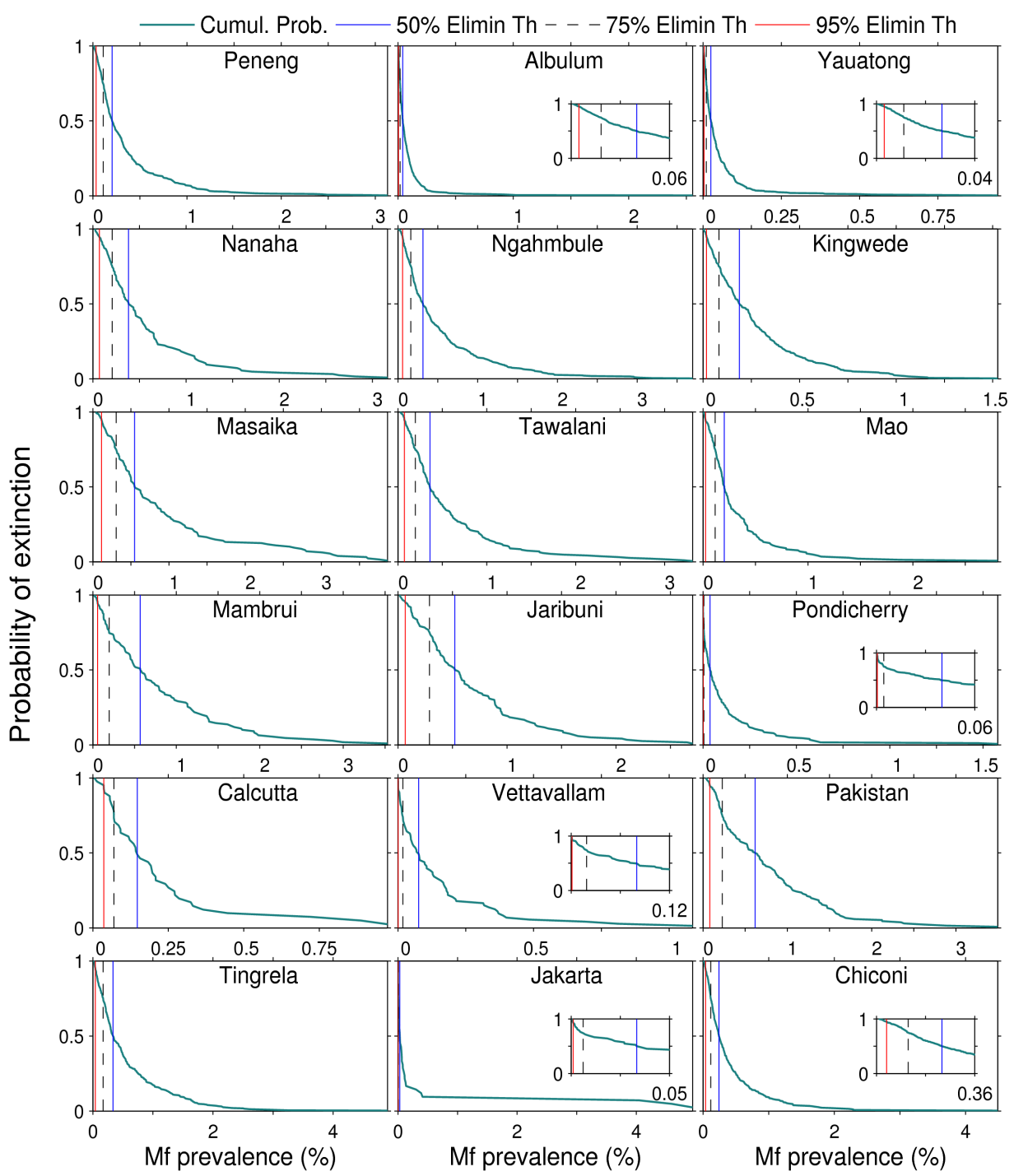


**Additional Figure S2 - Mf breakpoint threshold values for interrupting LF transmission at the prevailing ABR with different levels of elimination probabilities.** Given a distribution of threshold mf breakpoint values as estimated for each village by the BM fit of the LF model, we may use the complementary cumulative density function (CDF) of these estimates, as illustrated by the solid curves in the above figure, to calculate the exceedance risk of LF extinction for a given value of the mf breakpoint and thereby derive the probability of LF extinction arising when crossing below that breakpoint [19]. Here, we used this method to estimate three mf prevalence threshold values for which the elimination probabilities (EPs) are 50%, 75% and 95%, respectively. These values are shown by the three vertical dashed lines that intersect the cumulative probability curve in the figure, with the blue representing the mf value denoting a 50% probability of elimination, the black, a 75% EP, and the red, a 95% EP. Note that mf breakpoint values decline in value with increasing probabilities of elimination. The inset plots are given to clearly differentiate the three lines by in those settings (namely, ‘*Albulum*’, ‘*Yauatong*’, ‘*Pondicherry*’, ‘*Jakarta*’, and ‘*Chiconi*’), where they do not appear to be distinguishable from one another in the main plots.

Additional File 1


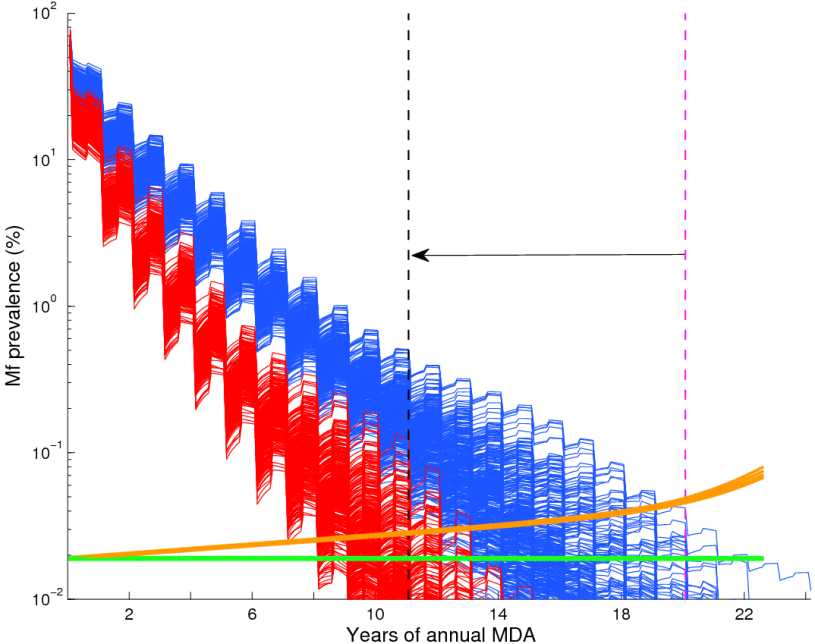


Additional Figure S3 - Illustration of the impact of vector control (VC) when used as a supplement to annual mass drug administration (MDA) on infection thresholds. The saw-toothed curves in the plot represent the modelled change in community-level mf prevalence (%) due to MDA-based interventions using the best-fit set of parameter vectors obtained from the Peneng study site of PNG. Note that the mf prevalence values on the y-axis are on a logarithmic scale. The red curves represent the declines in infection when LF intervention was simulated for annual MDA at 60% population-level coverage with 80% reduction in the mean annual biting rate due to VC, while the blue curves represent the corresponding change in mf prevalence for the same delivery of annual MDA but without VC. The orange and green horizontal lines depict the temporal evolution of elimination threshold values (in terms of % mf prevalence) over the period of each intervention: the orange line represents the case when MDA was supplemented by VC while the green line depicts the case when MDA was applied alone. The results show that as intervention progresses through time, VC will raise the LF elimination threshold by reducing the mosquito biting rate (see Additional Fig. 1). In the absence of VC, there will be no change in the elimination threshold during the intervention period. The two vertical dashed lines show the time points when the modelled mf prevalence for the two types of interventions had gone below the respective elimination threshold values for 90% of the best-fit parameter vectors. This indicates how increased effectiveness as well as raised elimination thresholds will allow the meeting of the goal of LF elimination earlier in the case of the MDA plus VC intervention in comparison with using MDA alone.

Additional File 1


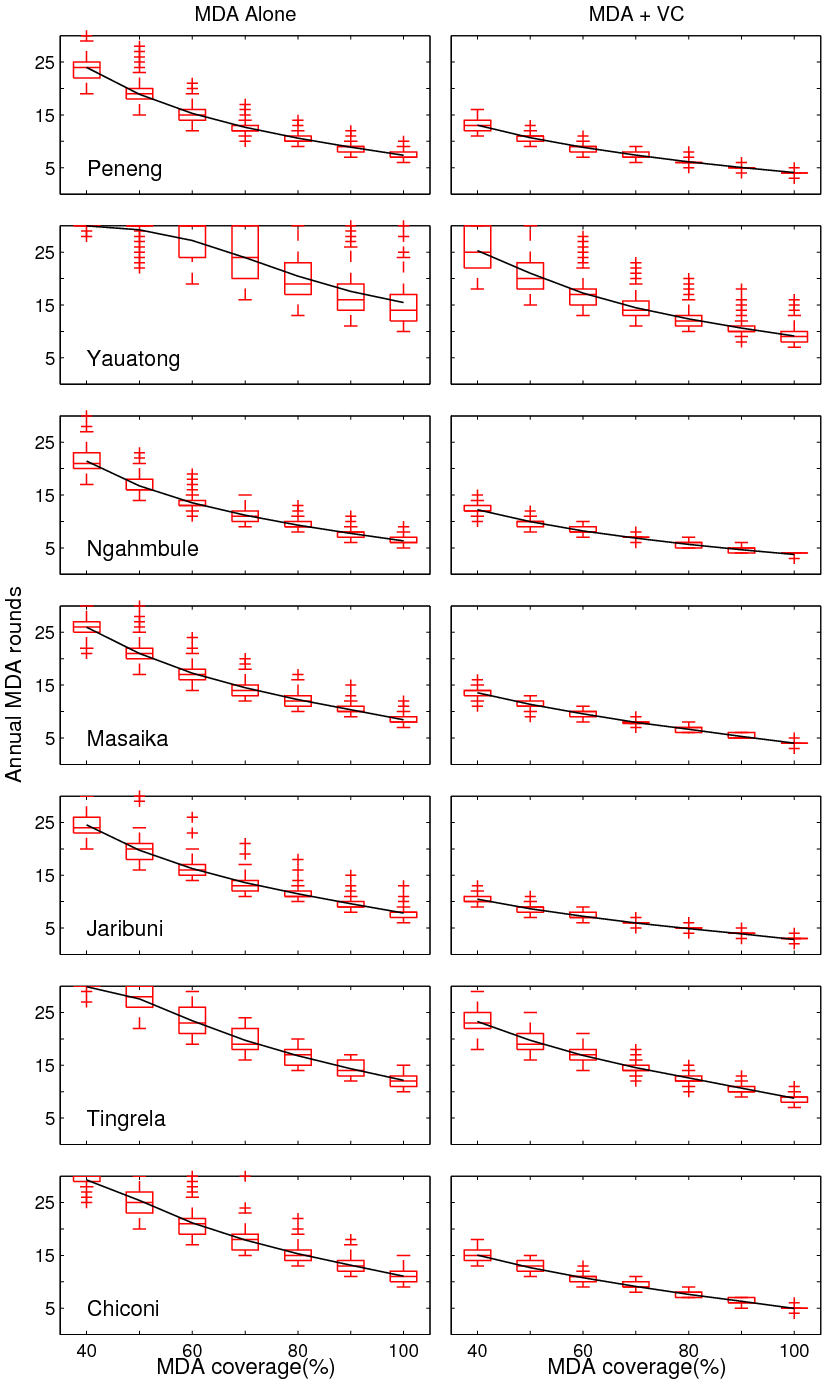


Additional Figure S4 - Variability in the impact of annual mass drug administration (MDA) in different LF endemic settings. These are shown for the remaining 7 anopheline study sites: top 3 sites from PNG while the remaining 4 ones from Africa. Supplemental use of vector control (VC) both reduces the number of years of interventions required to achieve LF interruption as well as variability in these years across all drug coverages from 40% to 100%. The results are shown for the transmission threshold with 95% elimination probability (EP). The results for the 50% and 75% EP thresholds show the same behaviour (data not shown). The results are from the model simulations for both LF intervention scenarios: MDA Alone and MDA + VC.

Additional File 1


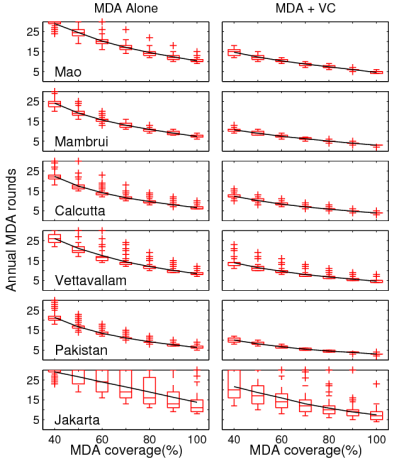


**Additional Figure S5 - Variability in the impact of annual MDA in different LF endemic settings.** These are shown for the remaining 6 culicine study sites: top 2 sites from Africa while the remaining 4 ones from the Southeast Asia. Everything else is as in **Additional Figure 4**.

References

1. Rajagopalan PK: **Population dynamics of Culex pipiens fatigans, the filariasis vector, in Pondicherry - influence of climate and environment.** Proc Ind Nat Science Acad 1980, **B46**:745-752.

2. Subramanian S, Manoharan A, Ramaiah KD, Das PK: **Rates of Acquisition and Loss of Wuchereria Bancrofti Infection in Culex Quinquefasciatus.** Am J Trop Med Hyg 1994, **51**(2):244-249.

3. Gambhir M, Michael E: **Complex ecological dynamics and eradicability of the vector borne macroparasitic disease, lymphatic filariasis.** PLoS One 2008, **3**(8):e2874.

4. Gambhir M, Bockarie M, Tisch D, Kazura J, Remais J, Spear R, Michael E: **Geographic and ecologic heterogeneity in elimination thresholds for the major vector-borne helminthic disease, lymphatic filariasis.** BMC Biol 2010, **8**:22.

5. Singh BK, Bockarie MJ, Gambhir M, Siba PM, Tisch DJ, Kazura J, Michael E: **Sequential modeling of the effects of mass drug treatments on Anopheline-mediated lymphatic filariasis infection in Papua New Guinea.** PLoS One 2013, **8**(6):e67004.

6. Hairston NG, de Meillon B: **On the inefficiency of transmission of Wuchereria bancrofti from mosquito to human host.** Bull World Health Organ 1968, **38**(6):935-941.

7. Ho BC, Ewert A: **Experimental transmission of filarial larvae in relation to feeding behaviour of the mosquito vectors.** Trans R Soc Trop Med Hyg 1967, **61**(5):663-666.

8. Vanamail P, Subramanian S, Das PK, Pani SP, Rajagopalan PK: **Estimation of fecundic life span of Wuchereria bancrofti from longitudinal study of human infection in an endemic area of Pondicherry (south India).** Indian J Med Res 1990, **91**(July):293-297.

9. Evans DB, Gelband H, Vlassoff C: **Social and economic factors and the control of lymphatic filariasis: A review.** Acta Trop 1993, **53**(1):1-26.

10. Ottesen EA, Ramachandran CP: **Lymphatic Filariasis Infection and Disease - Control Strategies.** Parasitol Today 1995, **11**(4):129-131.

11. Vanamail P, Ramaiah KD, Pani SP, Das PK, Grenfell BT, Bundy DAP: **Estimation of the fecund life span of Wuchereria bancrofti in an endemic area.** Trans R Soc Trop Med Hyg 1996, **90**(2):119-121.

12. Subramanian S, Krishnamoorthy K, Ramaiah KD, Habbema JDF, Das PK, Plaisier AP: **The relationship between microfilarial load in the human host and uptake and development of *Wuchereria bancrofti* microfilariae by *Culex quinquefasciatus*: a study under natural conditions.** Parasitology 1998, **116**(03):243-255.

13. Norman RA, Chan MS, Srividya A, Pani SP, Ramaiah KD, Vanamail P, Michael E, Das PK, Bundy DA: **EPIFIL: the development of an age-structured model for describing the transmission dynamics and control of lymphatic filariasis.** Epidemiol Infect 2000, **124**(3):529-41.

14. Subramanian S, Pani SP, Das PK, Rajagopalan PK: **Bancroftian Filariasis in Pondicherry, South India: 2. Epidemiological Evaluation of the Effect of Vector Control.** Epidemiol Infect 1989, **103**(3):693-702.

15. Das PK, Manoharan A, Subramanian S, Ramaiah KD, Pani SP, Rajavel AR, Rajagopalan PK: **Bancroftian Filariasis in Pondicherry, South India: Epidemiological Impact of Recovery of the Vector Population.** Epidemiol Infect 1992, **108**(3):483-493.

16. May RM: **Togetherness among Schistosomes: its effects on the dynamics of the infection.** Math Biosci 1977, **35**(3–4):301-343.

17. Duerr HP, Dietz K, Eichner M: **Determinants of the eradicability of filarial infections: a conceptual approach.** Trends Parasitol 2005, **21**(2):88-96.

18. Waller LA, Smith D, Childs JE, Real LA: **Monte Carlo assessments of goodness-of-fit for ecological simulation models.** Ecol Model 2003, **164**(1):49-63.

19. Reimer LJ, Thomsen EK, Tisch DJ, Henry-Halldin CN, Zimmerman PA, Baea ME, Dagoro H, Susapu M, Hetzel MW, Bockarie MJ, Michael E, Siba PM, Kazura JW: **Insecticidal Bed Nets and Filariasis Transmission in Papua New Guinea.** N Engl J Med 2013, **369**(8):745-753.
